# Lethal and Sub-lethal Implications of Sodium Chloride Exposure for Adult Unionid Mussel Species: *Eurynia dilatata* and *Lasmigona costata*

**DOI:** 10.1007/s00244-023-01006-0

**Published:** 2023-05-26

**Authors:** Erika A. Burton, Brian Atkinson, Joseph Salerno, Hufsa N. Khan, Ryan S. Prosser, Patricia L. Gillis

**Affiliations:** 1 Aquatic Contaminants Research Division, Environment and Climate Change Canada, Burlington, ON Canada; 2 Agriculture and Food Laboratory, University of Guelph, Guelph, ON Canada; 3 School of Environmental Sciences, University of Guelph, Guelph, ON Canada

## Abstract

The elevated use of salt as a de-icing agent on roads in Canada is causing an increase in the chloride concentration of freshwater ecosystems. Freshwater Unionid mussels are a group of organisms that are sensitive to increases in chloride levels. Unionids have greater diversity in North America than anywhere else on Earth, but they are also one of the most imperiled groups of organisms. This underscores the importance of understanding the effect that increasing salt exposure has on these threatened species. There are more data on the acute toxicity of chloride to Unionids than on chronic toxicity. This study investigated the effect of chronic sodium chloride exposure on the survival and filtering activity of two Unionid species (*Eurynia dilatata,* and *Lasmigona costata*) and assessed the effect on the metabolome in *L. costata* hemolymph. The concentration causing mortality after 28 days of exposure was similar for *E. dilatata* (1893 mg Cl^−^/L) and *L. costata* (1903 mg Cl^−^/L). Significant changes in the metabolome of the *L. costata* hemolymph were observed for mussels exposed to non-lethal concentrations. For example, several phosphatidylethanolamines, several hydroxyeicosatetraenoic acids, pyropheophorbide-a, and alpha-linolenic acid were significantly upregulated in the hemolymph of mussels exposed to 1000 mg Cl^−^/L for 28 days. While no mortality occurred in the treatment, elevated metabolites in the hemolymph are an indicator of stress.

**Supplementary Information:**

The online version contains supplementary material available at 10.1007/s00244-023-01006-0.

Salinization of freshwater systems is a growing global threat (Cañedo-Argüelles et al. 2013) with implications for ecosystem services and the health of freshwater biota at all trophic levels (Hintz and Relyea 2019). Secondary salinization of waterways via anthropogenic inputs originating from industrial effluent, oil and gas extraction, and road salt application (Elphick et al. 2011; Cañedo-Argüelles et al. 2013; Gillis et al. 2022; Hintz et al. 2022) has the potential to significantly affect freshwater systems, if not mitigated (Hintz and Relyea 2019). In southern Ontario (Canada), most salt inputs to freshwater systems have been attributed to road salt application (Evans and Frick 2001), with high concentrations linked to impervious surfaces, road density, and urbanized areas (Mazumder et al. 2021). The increased use of road salt on roadways in the last few decades has been responsible for the salinization of surface waters in temperate regions in Canada and the Northern USA (Jackson and Jobbagy 2005; Kaushal et al. 2005; Oswald et al. 2019). Chloride levels in many waterways exceed thresholds that can be harmful to freshwater organisms (Evans and Frick 2001; Jackson and Jobbagy 2005; Kaushal et al. 2005; Trowbridge et al. 2010) with some urban streams within the Greater Toronto Area (Ontario) reaching levels as high as 18,200 mg/L (OMOECC 2022) 28-fold greater than the guideline of 640 mg Cl^−^/L for acute chloride exposure to freshwater organisms (CCME 2011). In addition to the chloride spikes from road salt that occur in winter months, 40–90% of chloride inputs can be retained in soil and groundwater allowing for year-round release into freshwater systems and an increased baseline chloride concentration (Howard and Beck 1993; Kelly et al. 2008; Roy et al. 2015; Oswald et al. 2019). This creates the potential for year-round chronic stress for salt-sensitive biota (Mazumder et al. 2021).

Because of their heightened sensitivity to salt (Wang et al. 2007, 2018; Gillis 2011; Pandolfo et al. 2012; Blakeslee et al. 2013; Robertson et al. 2017; Salerno et al. 2018) and the levels reported in their habitats (Todd and Kaltenecker 2012; Patnode et al. 2015; Prosser et al. 2017b; Gillis et al. 2022), there are concerns that freshwater mussels including species at risk could be negatively affected by freshwater salinization (Todd and Kaltenecker 2012; Hintz and Relyea 2019). Canada has the lowest freshwater chloride guidelines worldwide (640 mg Cl^−^/L and 120 mg Cl^−^/L for acute and chronic exposure, respectively (CCME 2011), which has been attributed to the inclusion of freshwater Unionid mussels (Patnode et al. 2015; Hintz et al. 2022). However, in some waterbodies in Ontario, chloride guidelines are frequently exceeded.

With nearly 70% of North American freshwater mussel species listed as either endangered, threatened, or in decline, they are one of the most imperiled groups of aquatic organisms (Williams et al. 1993; Biggins et al. 1995; Bogan 2007) and are often more sensitive than standard test organisms (Wang et al. 2007) particularly to inorganic contaminants such as metals and salt (Wang et al. 2017). Recovery strategies produced under the Canadian Species at Risk Act indicate that the heightened sensitivity of freshwater mussels to environmental contaminants poses a threat to the recovery of at-risk species (COSEWIC 2015; Hintz et al. 2022).While the acute and chronic salt sensitivities of early life stage freshwater mussels have been demonstrated (Gillis 2011; Pandolfo et al. 2012; Prosser et al. 2017a; Wang et al. 2017, 2018; Salerno et al. 2018, 2020), the effect of salt on adult mussels, particularly the sub-lethal effects of longer-term exposure, is less understood despite potentially greater risk of exposure, as adult mussels can be exposed to chloride year-round.

Exposing adult mussels to hypersaline media have been shown to have negative consequences for mussel biochemistry, physiology, and behavior (Bertrand et al. 2017), and salt exposure can be lethal when mussels are exposed to water iso- or hyperosmotic to blood solute concentrations (Griffith 2017). Freshwater mussels maintain a low internal ion concentration compared with other freshwater invertebrates and have a limited capacity for active ion transport to maintain hypotonic body fluids (Dietz and Branton 1975; Dietz 1979; Griffith 2017; Hart et al. 2019), making their ability to respond to a hypersaline environment limited and energetically taxing (Hartmann et al. 2016). Physiological effects of salt exposure include the upregulation of cell membrane proteins (Robertson et al. 2017) and disruption of septate junctions (Griffith 2017). Mussels have also shown changes in energy use and storage (such as decreased condition index) when exposed to hypersaline solutions (Ciparis et al. 2019; Hart et al. 2019). Other changes in metabolism such as reduced oxygen consumption (Blakeslee et al. 2013) and reliance on anaerobic metabolism (Bertrand et al. 2017) have also been observed as sub-lethal responses to salt exposure.

Bertrand et al. (2017) attest that the reliance on anaerobic metabolism by mussels exposed to hypersaline water is related to the mussels’ change in shell closure behavior. Mussels change their filtering behavior in response to environmental stressors, closing their valves to avoid exposure to poor water quality and toxicants (Kramer et al. 1989; Hartmann et al. 2016). While preventing exposure to toxic substances over short periods, avoidance of filtering can lead to increased osmolarity, anoxia, and metabolic acidification, if the mussel is unable to exchange water and materials with its environment (Bertrand et al. 2017). Reduced filtration has been observed in freshwater mussels exposed to salt (Hartmann et al. 2016). In addition to whole organism endpoints of exposure such as filtering behavior and condition index that demonstrate stress to freshwater mussels at sub-lethal salt concentrations, investigations at the biochemical or metabolite level can reveal more subtle effects of salinity on an animal’s energy storage, cell integrity, and ion regulation. The biochemical endpoints can contribute to our understanding of the toxic mode of action in contaminant-stressed organisms.

A method to identify biochemical pathways that may be affected by exposure to a stressor is metabolomics. Metabolomic analysis, which is commonly used as a diagnostic tool in human and veterinary medicine, can reveal differences in metabolites, proteins, and electrolytes in an organism’s circulatory fluid, thereby providing insight into the health of the organism (Jones and Cheung 2007; Clish 2015). Several studies have investigated the use of metabolomics to understand the effects of toxicants in freshwater mollusks (Leonard et al. 2014a, 2014b; Roznere et al. 2014; Tufi et al. 2015, 2016), including the non-lethal approach of metabolite analysis in hemolymph (Gustafson et al. 2005). These studies have shown that targeted and non-targeted metabolomics hold potential in understanding the biochemical pathways that are affected by exposure to a toxicant and as an indicator of exposure to toxicants.

The purpose of this study was to evaluate acute and chronic effects of environmentally relevant chloride concentrations on the survival and metabolism of two freshwater mussel species: *Eurynia dilatata* and *Lasmigona costata*. Adult mussels were exposed to salt for 28 days to address the following research questions: (1) What concentration of salt is lethal to adult mussels? (2) Does salt exposure result in changes in filtering behavior and metabolite concentrations in mussel hemolymph? and (3) Can changes in the mussel metabolome identify biological pathways that are affected because of exposure to a chemical stressor?

## Methods

### ***Chloride Concentrations***

Mussels were exposed to six nominal concentrations of chloride (120, 250, 500, 1000, 2000, and 3750 mg Cl^−^/L) via a static renewal toxicity test. Exposures were completed in reconstituted moderately hard water (MHW) (USEPA 2002). The lowest concentration of chloride tested (120 mg/L) was based on the long-term Canadian Water Quality Guideline for the Protection of Aquatic Life (CCME 2011). Chloride concentrations in the exposure solutions were verified with a benchtop chloride probe (Orion chloride ion selective electrode 9617BNWP, Thermo Scientific, MA, USA) and were within 2.3% of the target chloride concentration (Supplementary Information (SI) Tables S1 to S3).

### *Mussel Selection and Retrieval*

Two mussel species were used for the exposure, *Eurynia dilatata* (Rafinesque 1820, formerly *Elliptio dilatata*, spike mussel) and *Lasmigona costata* (Rafinesque 1820, fluted-shell mussel). Approximately 70 individuals of each species were retrieved in July 2017 from a reference site on the Grand River (Ontario) with large populations of both species (43°29′40.7″N, 80°28′12.1″W). Individual adult mussels were chosen based on a size (*E. dilatata* mean length 89.4 mm, standard deviation (SD) 6.7; *L. costata* mean length 63.0 mm, SD 3.8) that fit well in the test vessels (108 mm diameter). Mussels were assessed for gravidity upon collection to ensure they were not actively reproducing, and approximate age of each mussel used in the exposure was determined by counting external annuli (Table S5).

Mussels were held in bins with continuous water flow (13° ± 2 C) for one week prior to salt exposure at Environment and Climate Change Canada’s (ECCC) Aquatic Life Research Facility (Burlington, ON) and fed 250 mL of an algae mixture (Reed Mariculture’s Instant Algae Shellfish Diet 1800 (12 mL) and Instant Algae Nano Diet 3600 (3 mL) in 450 mL of deionized water) twice daily within test vessels (Salerno et al. 2018).

### *Exposure Setup and Conditions*

Mussels were exposed following standard ASTM exposure guidelines for freshwater mussels (ASTM International 2013). Moderately hard water (MHW) was synthesized two days prior to the onset of mussel exposure and aerated. Sodium chloride solutions were prepared using MHW one day prior to test initiation. Test vessels consisted of a 1-L glass beaker containing 100 mL of rinsed aquarium sand (CaribSea Super Naturals Premium Aquarium Substrate) and 850 mL of exposure solution (Salerno et al. 2018). Beakers were affixed with a lid and airline and aerated at ambient temperature (~ 22 °C) for several hours before mussels were introduced. For both test species, each exposure concentration had five replicates and the controls (MHW) had ten replicates. An additional five replicate test vessels were created for the control, 120 mg Cl^−^/L, and 1000 mg Cl^−^/L treatments for *L. costata* for use in metabolomic analysis.

Prior to test initiation, the mussels that had been held at 13° ± 2 C were transferred to new bins and those bins were held at room temperature with continuous temperature monitoring to allow the mussels to acclimate to the ambient test temperature (~ 22 °C) over the course of 4 h. Each mussel species was arranged into three groups based on size (small, medium, and large), and mussels from each size group were sequentially added to the test vessels for a given concentration to randomize size. Water chemistry measurements (pH, dissolved oxygen, chloride, conductivity, temperature) were taken on the exposure stock solutions prior to 850 mL of solution being added to each vessel (Table S1) on day 0. Water chemistry (pH, dissolved oxygen, chloride, conductivity, temperature) was measured in the exposure solutions used in the renewals on days 7 and 14 (Table S1). Water chemistry was also measured in the exposure solutions removed from the test vessels on days 7 and 14 (Table S2). Total ammonia nitrogen was measured in the water removed from the test vessels on days 7, 14, and 28 by ECCC’s National Laboratory for Environmental Testing (NLET) (Burlington, ON) (Table S3), and total ammonia was measured between water changes using a colorimetric test kit (API Ammonia NH_3_/NH_4_^+^ Test Kit, Table S4).

Test vessels containing mussels were aerated and held under an 8-h light, 16-h dark cycle at ~ 22 °C for 28 days. On days 7 and 21, 80% of the exposure solutions were renewed in each test vessel. A full vessel change (i.e., new sand and 100% solution renewal) occurred on day 14.

### *Daily Observations and Mortality*

Each test vessel containing one mussel was fed 250 μL of an algal mix (Instant Algae Shellfish Diet 1800 and Instant Algae Nano Diet 3600® in 450 mL of deionized water with a total algal concentration of ~ 5.07 × 10^8^ cells/mL) twice daily. Mussels were observed for filtering behavior and mortality at each feeding. Mussels were assessed for filtration based on a binary criterion, either the mussel shell was open with siphons visible, or the mussel shell was closed with no visible siphons. If mussels were burrowed in the substrate, they were not considered filtering and were excluded from analysis for the period they remained burrowed. A mussel was considered functionally dead if its valves were gaping, and it did not close in response to a prodding stimulus. Deceased mussels were removed from the vessel, and water chemistry data were collected.

### ***Test Conclusion***

After 28 days, all surviving mussels were removed from their vessels. A sample of hemolymph (500–1000 µL) was collected from the posterior adductor muscle of 10 exposed *L. costata* from each of the control, 120 mg Cl^−^/L, and 1000 Cl^−^ mg/L treatments using a 5-mL syringe with a 21-gauge hypodermic needle. Samples were immediately flash frozen using dry ice and kept frozen at − 80 °C until metabolite extraction.

### *Extraction of Metabolites and Mass Spectrometry*

The method for extracting metabolites from freshwater mussel hemolymph was adapted from Bruce et al. (2009) and Want et al. (2013). A 300 µL subsample of hemolymph was added to a 2 mL microtube containing two glass beads (Fisher 3 mm, 11-312-10A Fisher Scientific) and 500 µL of chilled methanol. Tubes were then shaken on a vortex mixer for 90 min followed by centrifugation (16,000 g at 4 °C) for 10 min. A 0.5 mL aliquot of the supernatant was removed and filtered through a 0.22 µm PFTE syringe filter into an amber HPLC vial for analysis using high-performance liquid chromatography coupled to a quantitative time of flight mass spectrometer (HPLC-Q-TOF-MS).

The analytical method used was adapted and further optimized from Zhu et al. (2013) and Sawada and Hirai (2013). Analyses of the hemolymph extracts were performed on an Agilent 1200 HPLC liquid chromatograph interfaced with an Agilent UHD 6530 Q-TOF mass spectrometer. A C18 column (Agilent Poroshell 120, 50 mm × 4.6 mm 2.7 µm) was used for chromatographic separation with the following solvents: water with 0.1% formic acid (A) and acetonitrile with 0.1% formic acid (B). The mobile phase gradient was as follows: initial conditions were 2% B hold for 1 min and then increased to 100% B in 19 min followed by column wash at 100% B for 3.5-min and 10-min re-equilibration. The first 2 and last 5 min of gradient were sent to waste and not the spectrometer. The flow rate was maintained at 0.4 mL/min. The mass spectrometer electrospray capillary voltage was maintained at 4.0 kV and the drying gas temperature at 250 °C with a flow rate of 8 L/min. Nebulizer pressure was 30 psi, and the fragmentor was set to 160. Nitrogen was used as both nebulizing and drying gas and collision-induced gas. The mass-to-charge ratio was scanned across the m/z range of 50–1400 m/z in 4 GHz (extended dynamic range in both positive- and negative-ion mode). The instrument was externally calibrated with the ESI Tune Mix (Agilent Technologies). The sample injection volume was 30 µl. Only nine individuals were able to be analyzed in the 120 mg Cl^−^/L treatment for negative-ion mode due to insufficient volume of hemolymph available for extraction.

### ***Chloride Levels in Ontario Surface Water***

**Fig. 1:**
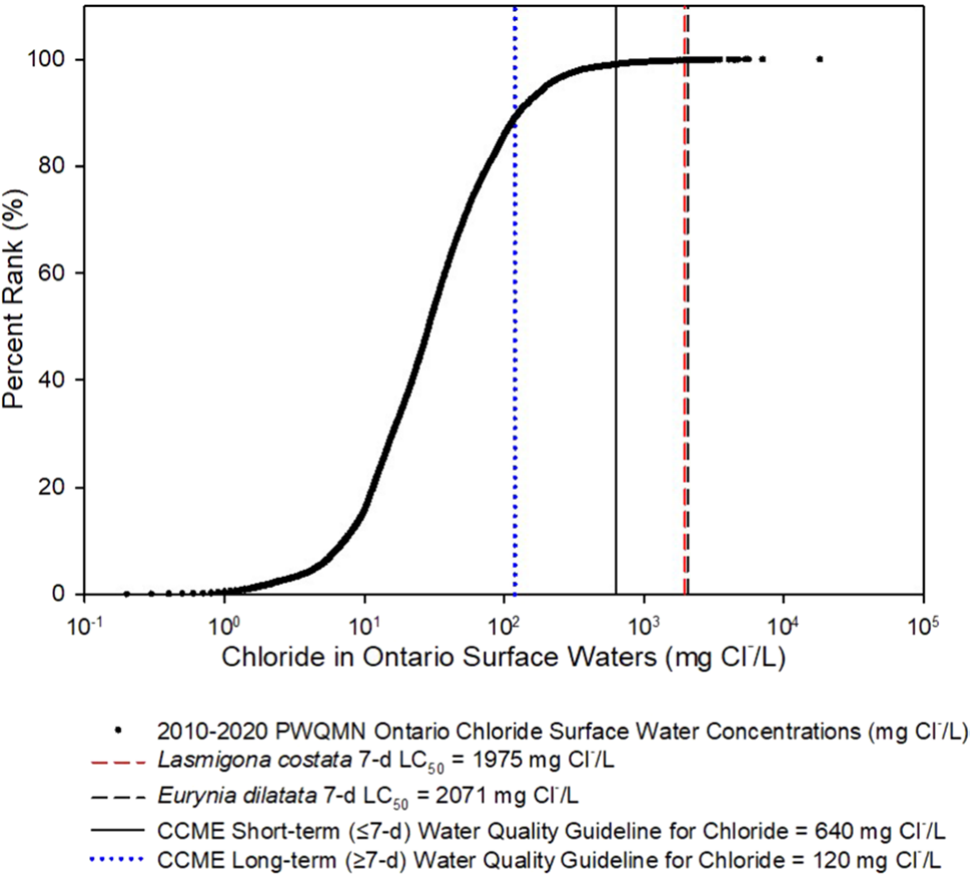
Cumulative frequency distribution of chloride concentrations in Ontario surface waters (2010–2020) using Provincial Water Quality Monitoring Network (PWQMN) data. The log concentration of chloride is reported on the x-axis, and the percent rank of the concentrations measured from 2010 to 2020 is on the y-axis. The dotted (blue) line represents the Canadian Council of Ministers of the Environment (CCME) long-term (120 mg Cl^−^/L) and short-term (640 mg Cl^−^/L) water quality guideline for the protection of aquatic life. The dashed lines represent the 7-d LC_50_ for *Lasmigona costata* (red) and *Eurynia dilatata* (black) from this study

Measured chloride levels in mussel habitats from the Ontario Provincial (Stream) Water Quality Monitoring Network (PWQMN) were consolidated. All published measured chloride levels in Ontario waterways from 2010 to 2020 were compiled and presented as a cumulative frequency distribution (Fig. 1).

### *Data Processing*

The dose–response curve (drc) package in R (Ritz et al. 2015; R Core Team 2021) was used to generate LC_50_ values for mussel mortality for each of 7, 14, 21, and 28 days of exposure as well as an associated 95% confidence interval. Mussels that were burrowed during an observation period were not included in the analysis of filtering. To determine significant differences in observed filtration time between treated mussels and controls, Chi-squared tests were performed in SigmaPlot (version 13.0). When assumptions of the Chi-squared test were violated, a Fisher exact test was completed.

Data processing for mass spectrometry was performed using Agilent Profinder software (version 10.0) and the batch recursive molecular feature extraction data mining algorithm. The resulting data matrix (30 positive-mode and 29 negative-mode observations; 1935 variables-positive mode, 280 variables-negative mode) was then exported for further data reduction. All entities (i.e., potential metabolites) were interpreted using Mass Profiler Professional (MPP) (version 15.1) software (Agilent Technologies) for statistical interpretation and compound identification. Statistical analysis was performed on the normalized data set using a one-way ANOVA with Benjamin–Hochberg testing correction with a *p* value set to 0.05. Following this, the entities were further reduced with setting a fold change cut-off of 2.0 in at least one condition. From the resulting refined data set, 121 positive and 81 negative entities were isolated. Interpretation of unknown entities was processed through the METLIN database and assigned a score from 0 to 100 that represents the probable match to that of curated spectral standards. To assess the variation in the hemolymph metabolome between control and treatment groups, a principal component analysis (PCA) was performed. Mass Profiler Professional was also used to perform cluster analysis and draw heatmaps representing the relative changes (upregulated/downregulated) in metabolites across the treatments. Agilent’s Pathways Architect (MPP, version 15.1) was used to identify curated biochemical pathways that contained metabolites that were identified as significantly different from the hemolymph of mussels among the three treatment groups.

## Results

### *Mussel Mortality*

**Fig. 2:**
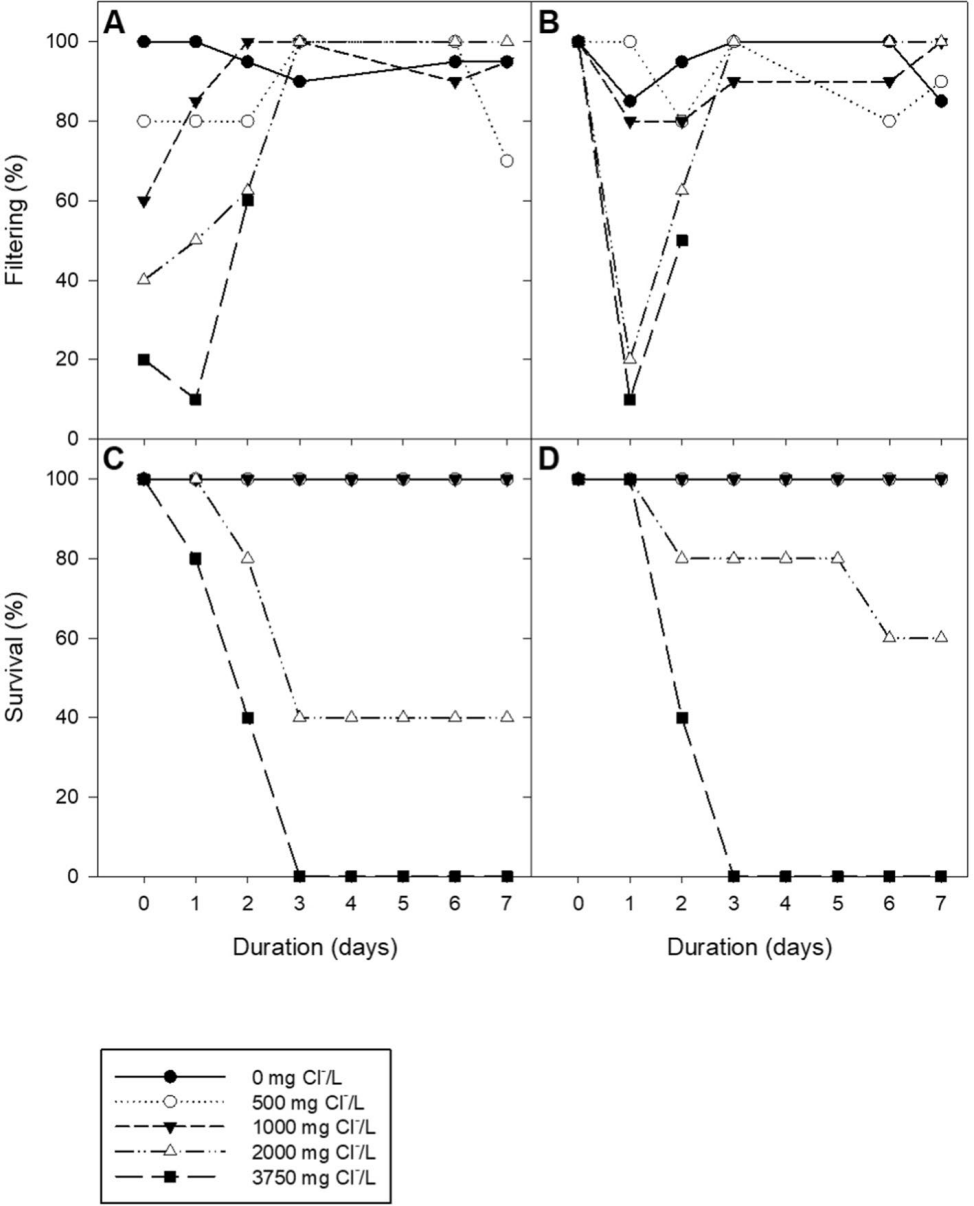
Overview of survival (%) and filtering activity (%) during the first 7 days of salt exposure with *Lasmigona costata* and *Eurynia dilatate.* Panel **A** and **B** depict the filtering activity (%) of *L. costata* and *E. dilatata*, respectively. Panel **C** and **D** depict the survival (%) of *L. costata* and *E. dilatata*, respectively. For clarity, only the control and four highest chloride treatments are shown. Chloride concentrations are nominal

While no mortality was observed in either species below 2000 mg Cl^−^/L, more than 75% of the mortality for *E. dilatata* and 100% of *L. costata* mortality in the higher concentrations (i.e., ≥ 2000 mg Cl^−^/L) occurred within the first 7 days of exposure (Fig. 2). All mussels in the highest treatment (3750 mg Cl^−^/L) died within the first 3 days of exposure. For *L. costata*, three of the five mussels in the 2000 mg/L treatment died in the first 3 days with no further mortality for the remainder of the test. In contrast, *E. dilatata* in the 2000 mg Cl^−^/L treatment died gradually during the 28-d exposure. LC50 estimates across time and species were similar (Table 1), with 7-day LC_50_s of 1975.4 mg Cl^−^/L for *L. costata* and 2070.7 mg Cl^−^/L for *E. dilatata*. LC_50_ estimates for chronic exposure (28 days) were also comparable with 1903.0 mg Cl^−^/L for *L. costata* and 1892.7 mg Cl^−^/L for *E. dilatata.*

**Table 1:** Summary of effect concentrations (mg Cl^−^/L) for adult mussel mortality (LC_50_)

Species	Mortality		
	Days	LC_50_	95% confidence interval
*L. costata*	7	1975.4	1319.8–2631.0
	14	2011.4	1557.7–2465.1
	21	2015.3	1509.9–2520.8
	28	1903.0	642.7–3163.3
*E. dilatata*	7	2070.7	1377.1–2764.3
	14	1884.4	607.0–3161.9
	21	1892.7	513.9–3271.6
	28	1892.7	513.9–3271.6

### *Filtration Behavior*

Differences in filtration behavior were most evident in the first 3 days of exposure, with *L. costata* demonstrating an overall pattern of reduced filtering with increasing salt concentration (Fig. 2 and Table S6). There were significant differences in observed filtration time between mussels in the control treatment compared with mussels in the two greatest chloride concentrations for both species of mussel (Table S7).

### ***Chloride Levels in Ontario***

The percentages of chloride measurements in Ontario’s waterways (i.e., PWQMN data) that exceeded a guideline or effect measure from this study are presented in Table 2. Eleven percent of the observations taken across a 10-year period exceeded the long-term CCME water guideline for the protection of aquatic life (120 mg Cl^−^/L), and 1% exceeded the short-term guideline of 640 mg Cl^−^/L. In addition, some concentrations of chloride measured in Ontario’s streams also exceeded the 7-day LC_50_ estimates (0.18% of observations for *L. costata* and 0.16% for *E. dilatata* (Table 2)) produced in this study.

**Table 2:** The number of chloride concentrations in Ontario surface waters (Provincial Water Quality Monitoring Network, 2010–2020) that exceeded effect measures for freshwater mussels (*Eurynia dilatata* and *Lasmigona costata*) or Canadian Council of Ministers of the Environment (CCME) guidelines

	7-day LC_50_		CCME guidelines		Total
	*L. costata*	*E. dilatata*	Short term	Long term	
# Detections	51	46	254	3112	28,384
% of Observations	0.2	0.2	0.9	11.0	

### *Metabolomics*

**Fig. 3:**
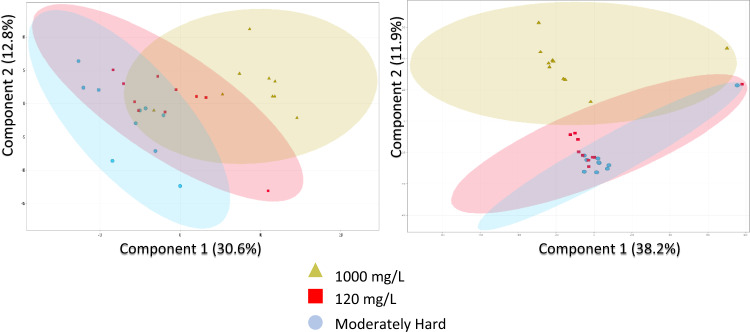
Principal component analysis plots created using the two principal components that describe the greatest amount of variation in metabolites among control (moderately hard water), 120 and 1000 mg Cl^−^/L exposure treatments. A 95% confidence ellipse has also been constructed for each treatment group. The number in brackets on each axis represents the percentage of variation in metabolome of hemolymph samples that is explained using each principal component. Metabolites identified using negative-mode ionization are presented in the biplot on the left, and metabolites identified using positive-mode ionization are presented in the biplot on the right. Each hemolymph sample in an exposure group is represented by a shape symbol

The metabolites found in the hemolymph of mussels exposed to two sub-lethal salt concentrations in this study were compared to those in control mussel hemolymph. PCA plots for both polarities showed significant separation between the annotated entities found in the 1000 mg Cl^−^/L group and the control group (Fig. 3). PCAs for both polarities show poor separation of annotated entities found between the 120 mg/L and the controls (Fig. 3). A total of 202 annotated entities were detected and considered statistically significant among the different treatment groups (Tables S8 and S9). These entities represented several classes of compounds including lipids, carbohydrates, alkaloids, carboxylic, and amino acids.

Overall, there was a strong trend of metabolites with upregulated fold changes at the 1000 mg Cl^−^/L treatment in both positive and negative modes versus the controls (Figs. S1 and S2). This aligns with the PCA plots showing that the metabolome of individuals in the 1000 mg/L was responding to the exposure (Fig. 3). When comparing the 120 mg Cl^−^/L treatment to the controls, there is evidence of significant fold changes in several metabolites in the hemolymph of individuals exposed to 120 mg Cl^−^/L in comparison with the controls.

## Discussion

### *Chloride Toxicity*

#### Mortality

In this study, mussels exposed to 2000 and 3750 mg Cl^−^/L showed morbidity within the first day of exposure, with 100% mortality in the highest treatment within 3 days of exposure. These results are comparable to the salt sensitivities of other (adult) freshwater mussel species. *Lampsilis siliquoidea* exposed to 3260 mg Cl^−^/L (Salerno et al. 2018) and *Elliptio complanata* exposed to 4000 mg Cl^−^/L for 7 days resulted in 100% mortality (Blakeslee et al. 2013). In contrast, no mortality was observed in *Popenaias popeii* (Texas hornshell), a species native to southern USA, exposed to 4000 mg Cl^−^/L (Hart et al. 2019).

In the current study, LC_50_ values for four different time points were generated, although the steep decline in survival between 1000 and 2000 mg Cl^−^/L treatments resulted in relatively greater confidence intervals for LC estimates (Table 1). Previous studies with adult mussels and sodium chloride also reported a steep decline in survival between concentrations of 2000 and 4000 mg Cl^−^/L (Blakeslee et al. 2013) and 2426 and 3033 mg Cl^−^/L (Hart et al. 2019). Both *L. costata* and *E. dilatata* showed similar sensitivity with considerable overlap in their LC_50_ confidence intervals (Table 1).

#### Filtration

Mussels are known to close their shell to avoid exposure to poor water quality and toxicants (Kramer et al. 1989; Bae and Park 2014; Patnode et al. 2015) or to reduce the need to osmoregulate, which may be energetically taxing (Hartmann et al. 2016). The salt-exposed mussels in the current study appear to have demonstrated this response (Fig. 2). *L costata* reduced filtration activity with increasing salt exposure, with significant reductions in the highest two concentrations compared with control mussels. A similar trend was observed in *E. dilatata* (Fig. 2). Hartmann et al. (2016) also found that mussels exposed to salt over long periods (up to 48 days) showed an overall reduction, as well as greater variation, in time spent filtering. Bertrand et al. (2017) suggested that changes in filtering behavior may be a signal of stress to the animals and can correspond with their eventual death.

### *Chloride Exposure in Natural Habitats*

Long-term water quality monitoring data (i.e., PWQMN) summarized herein demonstrate that between 2010 and 2020, there were numerous occasions when the chloride concentrations in Ontario surface waters surpassed both the Canadian Water Quality guidelines (CCME 2011) and the LC_50_ values derived in the current study for adult freshwater mussels (Fig. 1). This finding indicates that adult mussels could be experiencing lethal chloride toxicity in their natural habitats. It is also likely that the number of measurements exceeding harmful levels is underestimated because fewer measurements in the PWQMN program are taken in the winter months and thus the salt pulses associated with winter melt events could have been missed (Prosser et al. 2017b; Gillis et al. 2022).

### *Metabolomics*

#### Lipids

Lipids serve as an energy source and the raw materials for cell formation in mollusks (Sadjadi 2018). Roznere et al. (2014) identified a significant upregulation of lipids found in the hemolymph metabolome of *Amblema plicata* (three-ridge mussel) in response to captivity and food limitation, thereby demonstrating potential changes in the cellular lipid profiles of mollusks under stress. In this study, the levels of several long chain fatty acids (specifically phospholipids) increased in freshwater mussel hemolymph in response to chloride exposure. In total, 28 phosphatidylethanolamines (PEs), a key group of lipids in cellular membranes, were detected, of which 22 were found to have a significant upregulated fold change (> 2.0) in individuals exposed to 1000 mg Cl^−^/L compared with the control individuals. In addition, 14 of these PEs were also found to be upregulated in the individuals exposed to 120 mg Cl^−^/L. Moreover, several hydroxyeicosatetraenoic acids and their derivatives were found at higher levels in both the 120 and 1000 mg/L treatments in comparison with control mussels. This group of oxidized fatty acids can act as eicosanoids, which can be important in cell signaling. Several studies have observed elevated eicosanoid production in mollusk species that are under stress (Makoto et al. 1989; Stanley and Howard 1998; Pernet et al. 2007; Zhukova 2019). Pernet et al. (2007) observed an increase in fatty acids used in eicosanoid biosynthesis in the tissues of blue mussels *Mytilus edulis* and oysters *Crassostrea virginica* that were stressed by a rising temperature. The overall increase in profiles of these fatty acids could be an indication that the mussels are responding to the exposure of elevated salt concentrations to maintain homeostasis.

#### Energy and Metabolism Compounds

Several naturally occurring organic compounds and by-products of metabolism were found to be statistically different in the hemolymph of mussels exposed to chloride. Specifically, pyropheophorbide-a (PP-a), a chlorophyll degradation product that is egested as waste in mollusks, was found to be significantly upregulated in both the 120 and 1000 mg Cl^−^/L treatments compared with controls (Cartaxana et al. 2003). Pheophorbide concentrations (including PP-a) have been employed as indicators of feeding (consumption/egestion) rates in the fecal pellets of the marine gastropod, *Hydrobia ulvae* (Coelho et al. 2011). The observed increases in PP-a concentrations within the hemolymph in the current study could be a result of a reduction in the filtering activity of the mussels in response to the salt exposure. The mussels in the two greatest concentrations of this study spent significantly less time filtering than the mussels in the control test vessels (Fig. 2). Several other studies have observed a reduction in filtering behavior of mussels that have been exposed to contaminants, as was observed in this study (Hartmann et al. 2016; Chmist et al. 2019; Vijayavel and Kashian 2019). Hartmann et al. (2016) observed decreasing filtering activity in duck mussels (*Anodonta anatina)* exposed to pulses of de-icing salt. A reduction in filtering activity would reduce the clearance of waste, like the chlorophyll degradation product PP-a, from mussels. A reduction in the clearance of PP-a could cause an increase in the concentration measured in the hemolymph of mussels, as observed in this study.

#### Amino Acids and Peptides

Several amino acids and peptides in the hemolymph were found to be significantly different in mussels exposed to salt compared with unexposed mussels. Tumonoic acid A (TA-a), a naturally occurring amino acid derived from bacteria, was lower in the hemolymph of mussels exposed to 120 mg Cl^−^/L and was significantly downregulated at the 1000 mg Cl^−^/L treatment. This finding was unexpected as there does not appear to be any previous reports of TA-a in mollusks and its associated biological activity is unknown. Initial skepticism on the accuracy of the annotation of this entity was countered by the database score showing a strong match to TA-a and the metabolite database not returning any other potential matches at this mass range (Tables S8 and S9). The decreases in concentration of this metabolite could be a result of the decrease in filtering activity of the mussels, which would result in a decrease in the quantity of bacteria being consumed by individuals exposed to chloride.

Several short-chain peptides were identified, and all were found to be significantly upregulated in mussels exposed to the 1000 mg Cl^−^/L treatment compared with controls. The tripeptides Asn–Asp–Tyr and His–Lys–Gln were also significantly upregulated in mussels from the 1000 mg Cl^−^/L treatment. In addition, the dipeptide Met–Asn–OH was significantly upregulated in mussels exposed to the 1000 mg/L treatment with a 6000-fold change compared with controls. As with TA-a, these specific peptides have not been described in freshwater mussels, but they may be linked to a shift in metabolism and/or a response to oxidative stress. As shown by Bose et al. (2016) in the hemolymph of the land snail *Theba pisana* during aestivation (summer/dry season dormancy), a significant increase in amino acids and peptides could be indicative of an individual’s metabolism signaling a switch to energy storage and conservation in response to a stressor. A review conducted by Eghianruwa et al. (2019) also found that mollusks have a vast array of bioactive amino acids and peptides in the hemolymph relating to immune and antioxidant defense responses. While not found in this study, Schneeweiß and Renwrantz (1993) identified several formylated peptides which are a marker of immune system defense in response to chemical stressors.

#### Pathways Analysis

In this study, the search for pathways that related to the metabolites and were significantly different among the treatments in MPP included the pathway databases WikiPathways and BioCyc. Using the annotated pathways for *Danio rerio* (Zebrafish) did not produce any matches associated with the metabolites identified in this study. Using annotated pathways for *Homo sapiens*, the search revealed 42 total pathways that were impacted by salt exposure in the mussels (Tables S10 and S11). The pathways identified were mainly associated with the citric acid cycle (retinoic acid) and lipid metabolism (alpha-linolenic acid) (Tables S10 and S11).

Retinoic acid (RA) is a metabolite of vitamin A and is essential for cellular growth and development (McCaffery et al. 2006). Rothwell et al. (2017) identified that RA can modulate the function of networks in the central nervous system in mollusks. Specifically, they provided evidence that RA can reduce the transmission of signals across the synapse of excitatory neurons of mollusks (Rothwell et al. 2017). This metabolite was found to be upregulated in the individuals exposed to 1000 mg/L of chloride. This significant increase in RA could be related to an attempt to regulate the elevated osmotic pressure due to increased salt concentration, but this would require further investigation to confirm.

Linolenic and oleic derivatives were observed in both negative and positive modes. Alpha-linolenic acid (ALA) is a fatty acid found in plant and animal tissues. Zhukova (2019) reported that there is a relationship between fatty acid composition in mollusks and their interactions with the aquatic environment. Specifically, mollusks are not able to synthesize ALA, and as such, mollusks must derive ALA from their food. This metabolite was found to be significantly upregulated in the individuals exposed to 1000 mg/L of chloride. The elevated ALA could be a consequence of increased metabolism in response to salt exposure in comparison with the control and a decrease in egestion due to the mussels closing their valves in response to salt exposure.

A further investigation into the observed metabolites altered by exposure to chloride was conducted by manually searching the Kyoto Encyclopedia of Gene and Genomes (KEGG) database (Kanehisa and Goto 2000; Kanehisa 2019; Kanehisa et al. 2021). Currently, *Pomacea canaliculata* (golden apple snail) was the only mollusk with curated pathways in KEGG. Consequently, the *P. canaliculata* pathways were chosen as the most suitable for analyzing changes in the hemolymph metabolome of the mussels exposed to sodium chloride. A total of four pathways were identified that involve metabolites that were significantly different in *L. costata* exposed to sodium chloride compared with unexposed mussels. The pathways identified included alpha-linolenic acid metabolism, retinol metabolism, fatty acid biosynthesis, and steroid biosynthesis (Table S12). Alpha-linolenic acid metabolism, retinol metabolism, and fatty acid biosynthesis were also identified in the WikiPathways and BioCyc search, which corroborates that these pathways are affected when mussels are exposed to elevated concentrations of chloride. In terms of steroid biosynthesis, a cholesterol intermediate (zymosterol 1c) was found to be significantly down regulated in individuals in the 1000 mg Cl^−^/L treatment. Lange et al. (1991) reported that zymosterol is converted to cholesterol in the rough endoplasmic reticulum and is an important steroid in cell regulation. Kawashima et al. (2009) studied gastropods sterol composition in the gonads of *Cellana grata* and found that there is a significant difference in zymosterol based on sex. They stated that the physiological function of zymosterol remains unclear but could be involved with mitosis. The current study did not distinguish the sex of individuals, as *L. costata* are not externally sexually dimorphic. Further investigation will be needed to elucidate whether the findings correlate to differences in the sex of individuals or exposure to chloride.

Recent studies of the mollusk genome show progress is needed to unlock its full potential as a research tool for mollusks, and the same is true for transcriptomics and metabolomics (Luo et al. 2014; Gomes-dos-Santos et al. 2020). There are few metabolite and biochemical pathway databases that are curated specifically for mollusk species, whether freshwater, marine, or terrestrial. As a result, entities that are identified using non-target metabolomic analysis of mollusk tissues must be matched to metabolites, and subsequently biochemical pathways, from databases based on humans or other groups of species (fish, amphibians, other invertebrates). Notwithstanding this lack of mollusk-specific databases, many biochemical pathways are conserved across groups of eukaryotes and even more within deuterostomes (e.g., carbohydrate, amino acid, lipid, and nucleotide metabolism) (Peregrín-Alvarez et al. 2009). Conservation of biochemical pathways allows for some insight to be gained from non-targeted metabolomic analysis of tissue from mollusks that have been exposed to varying concentrations of a contaminant, as demonstrated by this study.

## Supplementary Information

Below is the link to the electronic supplementary material.

Supplementary file1 (DOCX 532 KB)

## Data Availability

Data are available in the supplemental information section and will be made available on Environment and Climate Change Canada’s Open Data portal.

## References

[CR1] ASTM International (2013). Standard guide for conducting laboratory toxicity tests with freshwater mussels.

[CR2] Bae M-J, Park Y-S (2014). Biological early warning system based on the responses of aquatic organisms to disturbances: a review. Sci Total Environ.

[CR3] Bertrand C, Devin S, Mouneyrac C, Giambérini L (2017). Eco-physiological responses to salinity changes across the freshwater-marine continuum on two euryhaline bivalves: *Corbicula fluminea* and *Scrobicularia plana*. Ecol Ind.

[CR4] Biggins R, Neves R, Dohner C (1995). Draft national strategy for the conservation of native freshwater mussels.

[CR5] Blakeslee CJ, Galbraith HS, Robertson LS, St John White  B (2013). The effects of salinity exposure on multiple life stages of a common freshwater mussel, *Elliptio complanata*. Environ Toxicol Chem.

[CR6] Bogan AE (2007). Global diversity of freshwater mussels (Mollusca, Bivalvia) in freshwater Freshwater animal diversity assessment.

[CR7] Bose U, Centurion E, Hodson M, Shaw P, Storey KB, Cummins SF (2016). Global metabolite analysis of the land snail *Theba pisana* hemolymph during active and aestivated states. Comp Biochem Physiol D: Genom Proteom.

[CR8] Bruce SJ, Tavazzi I, Parisod V, Rezzi S, Kochhar S, Guy PA (2009). Investigation of human blood plasma sample preparation for performing metabolomics using ultrahigh performance liquid chromatography/mass spectrometry. Anal Chem.

[CR9] Cañedo-Argüelles M, Kefford BJ, Piscart C, Prat N, Schäfer RB, Schulz C-J (2013). Salinisation of rivers: an urgent ecological issue. Environ Pollut.

[CR10] Cartaxana P, Jesus B, Brotas V (2003). Pheophorbide and pheophytin a-like pigments as useful markers for intertidal microphytobenthos grazing by *Hydrobia ulvae*. Estuar Coast Shelf Sci.

[CR11] CCME (2011) Canadian water quality guidelines : chloride ion. scientific criteria document. Canadian Council of Ministers of the Environment, Winnipeg, MB, Canada. https://ccme.ca/fr/res/2011-chloride-ceqg-scd-1460-en.pdf

[CR12] Chmist J, Szoszkiewicz K, Drożdżyński D (2019). Behavioural responses of *Unio tumidus* freshwater mussels to pesticide contamination. Arch Environ Contam Toxicol.

[CR13] Ciparis S, Rhyne G, Stephenson T (2019). Exposure to elevated concentrations of major ions decreases condition index of freshwater mussels: comparison of metrics. Freshw Mollusk Biol Conserv.

[CR14] Clish CB (2015). Metabolomics: an emerging but powerful tool for precision medicine. Cold Spring Harb Mol Case Stud.

[CR80] Coelho H, Cartaxana P, Brotas V, Queiroga H, Serôdio J (2011) Pheophorbide a in Hydrobia ulvae faecal pellets as a measure of microphytobenthos ingestion: variation over season and period of day. Aquat Biol 13:119–126

[CR15] COSEWIC (2015) COSEWIC assessment and status report on the Rainbow mussel *Villosa iris* in Canada. Committee on the Status of Endangered Wildlife in Canada, Ottawa, ON, Canada. https://www.canada.ca/en/environment-climate-change/services/species-risk-public-registry.html

[CR16] Dietz TH (1979). Uptake of sodium and chloride by freshwater mussels. Can J Zool.

[CR17] Dietz T, Branton W (1975). Ionic regulation in the freshwater mussel, *Ligumia subrostrata* (Say). J Comp Physiol.

[CR18] Eghianruwa QA, Osoniyi OR, Maina N, Wachira S (2019). Bioactive peptides from marine molluscs-a review. Int J Biochem Res Rev.

[CR19] Elphick JR, Bergh KD, Bailey HC (2011). Chronic toxicity of chloride to freshwater species: effects of hardness and implications for water quality guidelines. Environ Toxicol Chem.

[CR20] Evans M, Frick C (2001). The effects of road salts on aquatic ecosystem environment and climate change Canada.

[CR21] Gillis PL (2011). Assessing the toxicity of sodium chloride to the glochidia of freshwater mussels: implications for salinization of surface waters. Environ Pollut.

[CR22] Gillis PL, Salerno J, McKay VL, Bennett CJ, Lemon KL, Rochfort QJ, Prosser RS (2022). Salt-laden winter runoff and freshwater mussels; assessing the effect on early life stages in the laboratory and wild mussel populations in receiving waters. Arch Environ Contam Toxicol.

[CR23] Gomes-dos-Santos A, Lopes-Lima M, Castro LFC, Froufe E (2020). Molluscan genomics: the road so far and the way forward. Hydrobiologia.

[CR24] Griffith MB (2017). Toxicological perspective on the osmoregulation and ionoregulation physiology of major ions by freshwater animals: teleost fish, Crustacea, aquatic insects, and Mollusca. Environ Toxicol Chem.

[CR25] Gustafson LL, Stoskopf MK, Bogan AE, Showers W, Kwak TJ, Hanlon S, Levine JF (2005). Evaluation of a nonlethal technique for hemolymph collection in *Elliptio complanata*, a freshwater bivalve (Mollusca: Unionidae). Dis Aquat Org.

[CR26] Hart MA, Miller TD, Randklev CR (2019). Salinity tolerance of a rare and endangered unionid mussel, *Popenaias popeii* (*Texas Hornshell*) and its implications for conservation and water management. Ecotoxicol Environ Saf.

[CR27] Hartmann JT, Beggel S, Auerswald K, Stoeckle BC, Geist J (2016). Establishing mussel behavior as a biomarker in ecotoxicology. Aquat Toxicol.

[CR28] Hintz WD, Relyea RA (2019). A review of the species, community, and ecosystem impacts of road salt salinisation in fresh waters. Freshw Biol.

[CR29] Hintz WD, Arnott SE, Symons CC, Greco DA, McClymont A, Brentrup JA, Cañedo-Argüelles M, Derry AM, Downing AL, Gray DK (2022). Current water quality guidelines across North America and Europe do not protect lakes from salinization. Proc Natl Acad Sci.

[CR30] Howard KW, Beck PJ (1993). Hydrogeochemical implications of groundwater contamination by road de-icing chemicals. J Contam Hydrol.

[CR31] Jackson RB, Jobbagy EG (2005). From icy roads to salty streams. Proc Natl Acad Sci.

[CR32] Jones OA, Cheung VL (2007). An introduction to metabolomics and its potential application in veterinary science. Comp Med.

[CR33] Kanehisa M (2019). Toward understanding the origin and evolution of cellular organisms. Protein Sci.

[CR34] Kanehisa M, Goto S (2000). KEGG: kyoto encyclopedia of genes and genomes. Nucleic Acids Res.

[CR35] Kanehisa M, Furumichi M, Sato Y, Ishiguro-Watanabe M, Tanabe M (2021). KEGG: integrating viruses and cellular organisms. Nucleic Acids Res.

[CR36] Kaushal SS, Groffman PM, Likens GE, Belt KT, Stack WP, Kelly VR, Band LE, Fisher GT (2005). Increased salinization of fresh water in the Northeastern United States. Proc Natl Acad Sci.

[CR37] Kawashima H, Ohnishi M, Ogawa S (2009). Differences in sterol composition between male and female gonads of dominant limpet species. Lipids.

[CR38] Kelly VR, Lovett GM, Weathers KC, Findlay SE, Strayer DL, Burns DJ, Likens GE (2008). Long-term sodium chloride retention in a rural watershed: legacy effects of road salt on streamwater concentration. Environ Sci Technol.

[CR39] Kramer KJ, Jenner HA, de Zwart D (1989). The valve movement response of mussels: a tool in biological monitoring. Hydrobiologia.

[CR40] Lange Y, Echevarria F, Steck TL (1991). Movement of zymosterol, a precursor of cholesterol, among 3 membranes in human fibroblasts. J Biol Chem.

[CR41] Leonard JA, Cope WG, Barnhart MC, Bringolf RB (2014). Metabolomic, behavioral, and reproductive effects of the aromatase inhibitor fadrozole hydrochloride on the unionid mussel *Lampsilis fasciola*. Gen Comp Endocrinol.

[CR42] Leonard JA, Cope WG, Barnhart MC, Bringolf RB (2014). Metabolomic, behavioral, and reproductive effects of the synthetic estrogen 17 α-ethinylestradiol on the unionid mussel *Lampsilis fasciola*. Aquat Toxicol.

[CR43] Luo Y, Li C, Landis AG, Wang G, Stoeckel J, Peatman E (2014). Transcriptomic profiling of differential responses to drought in two freshwater mussel species, the giant floater *Pyganodon grandis* and the pondhorn *Uniomerus tetralasmus*. PLoS ONE.

[CR44] Makoto O, Masazumi N, Tadashi N (1989). Involvement of prostaglandins in the spawning of the scallop, Patinopecten yessoensis. Comp Biochem Physiol Part C: Comp Pharmacol.

[CR45] Mazumder B, Wellen C, Kaltenecker G, Sorichetti RJ, Oswald CJ (2021). Trends and legacy of freshwater salinization: untangling over 50 years of stream chloride monitoring. Environ Res Lett.

[CR46] McCaffery P, Zhang J, Crandall JE (2006). Retinoic acid signaling and function in the adult hippocampus. J Neurobiol.

[CR47] OMOECC (2022). Provincial water quality monitoring data for chloride—2000 to 2022.

[CR48] Oswald CJ, Giberson G, Nicholls E, Wellen C, Oni S (2019). Spatial distribution and extent of urban land cover control watershed-scale chloride retention. Sci Total Environ.

[CR49] Pandolfo TJ, Cope WG, Young GB, Jones JW, Hua D, Lingenfelser SF (2012). Acute effects of road salts and associated cyanide compounds on the early life stages of the unionid mussel *Villosa iris*. Environ Toxicol Chem.

[CR50] Patnode KA, Hittle E, Anderson RM, Zimmerman L, Fulton JW (2015). Effects of high salinity wastewater discharges on unionid mussels in the Allegheny River, Pennsylvania. J Fish Wildl Manag.

[CR51] Peregrín-Alvarez JM, Sanford C, Parkinson J (2009). The conservation and evolutionary modularity of metabolism. Genome Biol.

[CR52] Pernet F, Tremblay RJ, Comeau L, Guderley H (2007). Temperature adaptation in two bivalve species from different thermal habitats: energetics and remodelling of membrane lipids. J Exp Biol.

[CR53] Prosser R, Gillis P, Holman E, Schissler D, Ikert H, Toito J, Gilroy E, Campbell S, Bartlett A, Milani D (2017). Effect of substituted phenylamine antioxidants on three life stages of the freshwater mussel *Lampsilis siliquoidea*. Environ Pollut.

[CR54] Prosser R, Rochfort Q, McInnis R, Exall K, Gillis P (2017). Assessing the toxicity and risk of salt-impacted winter road runoff to the early life stages of freshwater mussels in the Canadian province of Ontario. Environ Pollut.

[CR55] R Core Team (2021) R: A languate and environment for statistical computing. R Foundation for Statistical Computing. https://www.r-project.org/

[CR56] Ritz C, Baty F, Streibig JC, Gerhard D (2015). Dose-response analysis using R. PLoS ONE.

[CR57] Robertson LS, Galbraith HS, Iwanowicz D, Blakeslee CJ, Cornman RS (2017). RNA sequencing analysis of transcriptional change in the freshwater mussel *Elliptio complanata* after environmentally relevant sodium chloride exposure. Environ Toxicol Chem.

[CR58] Rothwell CM, de Hoog E, Spencer GE (2017). The role of retinoic acid in the formation and modulation of invertebrate central synapses. J Neurophysiol.

[CR59] Roy JW, McInnis R, Bickerton G, Gillis PL (2015). Assessing potential toxicity of chloride-affected groundwater discharging to an urban stream using juvenile freshwater mussels (*Lampsilis siliquoidea*). Sci Total Environ.

[CR60] Roznere I, Watters GT, Wolfe BA, Daly M (2014). Nontargeted metabolomics reveals biochemical pathways altered in response to captivity and food limitation in the freshwater mussel *Amblema plicata*. Comp Biochem Physiol D: Genom Proteom.

[CR61] Sadjadi N (2018). Chemical ecology of biocompounds in molluscs biological resources of water.

[CR62] Salerno J, Gillis PL, Bennett CJ, Sibley PK, Prosser RS (2018). Investigation of clearance rate as an endpoint in toxicity testing with freshwater mussels (Unionidae). Ecotoxicol Environ Saf.

[CR63] Salerno J, Gillis P, Khan H, Burton E, Deeth L, Bennett C, Sibley P, Prosser R (2020). Sensitivity of larval and juvenile freshwater mussels (unionidae) to ammonia, chloride, copper, potassium, and selected binary chemical mixtures. Environ Pollut.

[CR64] Sawada Y, Hirai MY (2013). Integrated LC-MS/MS system for plant metabolomics. Comput Struct Biotechnol J.

[CR65] Schneeweiß H, Renwrantz L (1993). Analysis of the attraction of haemocytes from *Mytilus edulis* by molecules of bacterial origin. Dev Comp Immunol.

[CR66] Stanley DW, Howard RW (1998). The biology of prostaglandins and related eicosanoids in invertebrates: cellular, organismal and ecological actions. Am Zool.

[CR67] Todd AK, Kaltenecker MG (2012). Warm season chloride concentrations in stream habitats of freshwater mussel species at risk. Environ Pollut.

[CR68] Trowbridge PR, Kahl JS, Sassan DA, Heath DL, Walsh EM (2010). Relating road salt to exceedances of the water quality standard for chloride in New Hampshire streams. Environ Sci Technol.

[CR69] Tufi S, Stel JM, de Boer J, Lamoree MH, Leonards PE (2015). Metabolomics to explore imidacloprid-induced toxicity in the central nervous system of the freshwater snail *Lymnaea stagnalis*. Environ Sci Technol.

[CR70] Tufi S, Wassenaar PN, Osorio V, de Boer J, Leonards PE, Lamoree MH (2016). Pesticide mixture toxicity in surface water extracts in snails (*Lymnaea stagnalis*) by an in vitro acetylcholinesterase inhibition assay and metabolomics. Environ Sci Technol.

[CR71] USEPA (2002). Methods for measuring the acute toxicity of effluents and receiving waters to freshwater and marine organisms.

[CR72] Vijayavel K, Kashian DR (2019). Toxic effect and physiological disruption of sodium phosphate to the quagga mussel (*Dreissena bugensis*). Environ Sci Pollut Res.

[CR73] Wang N, Ingersoll CG, Hardesty DK, Ivey CD, Kunz JL, May TW, Dwyer FJ, Roberts AD, Augspurger T, Kane CM (2007). Acute toxicity of copper, ammonia, and chlorine to glochidia and juveniles of freshwater mussels (Unionidae). Environ Toxicol Chem.

[CR74] Wang N, Ivey CD, Ingersoll CG, Brumbaugh WG, Alvarez D, Hammer EJ, Bauer CR, Augspurger T, Raimondo S, Barnhart MC (2017). Acute sensitivity of a broad range of freshwater mussels to chemicals with different modes of toxic action. Environ Toxicol Chem.

[CR75] Wang N, Ivey CD, Dorman RA, Ingersoll CG, Steevens J, Hammer EJ, Bauer CR, Mount DR (2018). Acute toxicity of sodium chloride and potassium chloride to a unionid mussel (*Lampsilis siliquoidea*) in water exposures. Environ Toxicol Chem.

[CR76] Want EJ, Masson P, Michopoulos F, Wilson ID, Theodoridis G, Plumb RS, Shockcor J, Loftus N, Holmes E, Nicholson JK (2013). Global metabolic profiling of animal and human tissues via UPLC-MS. Nat Protoc.

[CR77] Williams JD, Warren ML, Cummings KS, Harris JL, Neves RJ (1993). Conservation status of freshwater mussels of the United States and Canada. Fisheries.

[CR78] Zhu Z-J, Schultz AW, Wang J, Johnson CH, Yannone SM, Patti GJ, Siuzdak G (2013). Liquid chromatography quadrupole time-of-flight mass spectrometry characterization of metabolites guided by the METLIN database. Nat Protoc.

[CR79] Zhukova NV (2019). Fatty acids of marine mollusks: impact of diet bacterial symbiosis and biosynthetic potential. Biomolecules.

