# Supplementary material for: Lethal and Sub-lethal Implications of Sodium Chloride Exposure for Adult Unionid Mussel Species: Eurynia dilatata and Lasmigona costata
Source: Arch Environ Contam Toxicol. 2023 May 26;85(1):1–12. doi: 10.1007/s00244-023-01006-0 (PMC10374710; doi:10.1007/s00244-023-01006-0)
Supplement: Supplementary file 1 — Supplementary file1 (DOCX 532 KB) [file 244_2023_1006_MOESM1_ESM.docx]

**SUPPLEMENTARY INFORMATION**

**Lethal and sub-lethal implications of sodium chloride exposure for adult Unionid mussel** **species; *Eurynia dilatata* and *Lasmigona costata***

Erika A Burton^a^, Brian Atkinson^b^, Joseph Salerno^a^, Hufsa N Khan^a^, Ryan S Prosser^c^, Patricia L Gillis*^a^

^a^Aquatic Contaminants Research Division, Environment and Climate Change Canada, Burlington, ON, Canada

^b^Agriculture and Food Laboratory, University of Guelph, Guelph, ON, Canada

^c^School of Environmental Sciences, University of Guelph, Guelph, ON, Canada

* Corresponding author: Patty.Gillis@ec.gc.ca

**Table S1**. Mean water quality parameters measured in exposure solution for each treatment made on days 0, 7, and 14 (n = 3). The number in brackets is a single standard deviation. For the 3750 mg Cl^-^/L treatment, the water was only measured in the exposure solution made at day 0, as all the mussels in this treatment died in the first seven days of the exposure.

| **Nominal Chloride Treatment (mg/L)** | **Chloride (mg/L)** | **Temperature (°C)** | **Conductivity (mS/cm)** | **Dissolved Oxygen (mg/L)** | **pH** |
| --- | --- | --- | --- | --- | --- |
| 0 | 1.3 (1.2) | 23.3 (0.8) | 0.33 (0.004) | 8.6 (0.2) | 8.2 (0.03) |
| 120 | 118.6 (8.3) | 22.6 (0.5) | 0.76 (0.009) | 8.6 (0.2) | 8.2 (0.02) |
| 250 | 247.3 (6.0) | 22.5 (0.5) | 1.2 (0.02) | 8.7 (0.3) | 8.2 (0.02) |
| 500 | 517.8 (22.7) | 22.4 (0.4) | 2.1 (0.02) | 8.5 (0.2) | 8.2 (0.03) |
| 1000 | 1033.7 (20.3) | 22.5 (0.4) | 3.7 (0.04) | 8.6 (0.4) | 8.2 (0.02) |
| 2000 | 2070.8 (44.6) | 22.6 (0.3) | 7.2 (0.5) | 8.6 (0.5) | 8.1 (0.03) |
| 3750 | 3792 | 22.7 | 15.6 | 8.3 | 8.1 |

**Table S2**. Mean water quality parameters measured in composite exposure solutions for each treatment made post exposure. Composite samples were analyzed for each species on day 7 and 14 (n=4). The number in brackets is a single standard deviation. For the 3750 mg Cl^-^/L treatment no data was collected as no mussels survived to day 7.

| **Nominal Chloride Treatment (mg/L)** | **Chloride (mg/L)** | **Temperature (°C)** | **Conductivity (mS/cm)** | **Dissolved Oxygen (mg/L)** | **pH** |
| --- | --- | --- | --- | --- | --- |
| 0 | 5.4 (3.2) | 22.7 (0.3) | 0.38 (0.03) | 8.4 (0.4) | 8.1 (0.1) |
| 120 | 118 (6.5) | 22.9 (0.5) | 0.8 (0.05) | 8.3 (0.2) | 8 (0.3) |
| 250 | 253.5 (22.3) | 22.6 (0.7) | 1.3 (0.08) | 8.4 (0.3) | 8 (0.1) |
| 500 | 524.5 (60.2) | 22.9 (0.5) | 2.1 (0.1) | 8.5 (0.2) | 8.1 (0.03) |
| 1000 | 1100 (49.7) | 23 (0.4) | 3.7 (0.2) | 8.4 (0.4) | 8.2 (0.1) |
| 2000 | 2082.5 (177.5) | 23 (0.4) | 6.1 (0.8) | 9.5 (2) | 8.2 (0.1) |

**Table S3**. Concentrations of ammonia measured in exposure solutions collected from test vessels before each solution change at day 7, 14, and 28. During water and vessel changes, solutions from each test vessel within a treatment were retained, combined, and submitted to Environment and Climate Change Canada’s National Laboratory for Environmental Testing for analysis of ammonia concentrations. The treatment with the greatest concentration of chloride was not included as the mussels died in the first seven days of exposure, making a water and vessel change unnecessary.

|  |  | **Measured ammonia (mg/L)** | | | |
| --- | --- | --- | --- | --- | --- |
| **Species** | **Nominal Chloride Treatment (mg Cl^-^/L)** | **Day 7** | **Day 14** | **Day 28** | **Average** |
| *Lasmigona costada* | 0 | 1.46 | 0.295 | 0.193 | 0.65 |
|  | 120 | 1.4 | 0.485 | 1.01 | 0.97 |
|  | 250 | 1.18 | 0.25 | 0.354 | 0.59 |
|  | 500 | 1.15 | 0.355 | 0.168 | 0.56 |
|  | 1000 | 2.86 | 0.675 | 0.371 | 1.30 |
|  | 2000 | 2.5 | 0.23 | 0.062 | 0.93 |
| *Eurynia dilatata* | 0 | 1.74 | 0.07 | 0.056 | 0.62 |
|  | 120 | 1.09 | 0.105 | 0.103 | 0.43 |
|  | 250 | 1.87 | 0.14 | 0.175 | 0.73 |
|  | 500 | 1.82 | 0.145 | 0.247 | 0.74 |
|  | 1000 | 2.3 | 1.03 | 0.706 | 1.35 |
|  | 2000 | 2.5 | 0.56 | 0.067 | 1.04 |

**Table S4**. Average ammonia concentrations by treatment in test beakers during exposure. Concentrations of total ammonia were measured using a colorimetric assay (API Ammonia Test Kit) which measures ammonia at 0, 0.25, 0.50, 1-8 ppm total ammonia. Standard deviation is presented in brackets. The number of *E. dilitata* from the 2000 mg CL^-^/L differ with each sampling date as individuals died.

|  | **Treatment (mg Cl^-^/L)** |  | **Average Total Ammonia Concentration (mg/L)** | | |
| --- | --- | --- | --- | --- | --- |
| **Species** |  | **N** | **Day 3** | **Day 10** | **Day 17** |
| *L. costata* | MHW | 10 | 0.45 (0.23) | 0.33 (0.41) | 0.35 (0.13) |
|  | 120 | 10 | 0.55 (0.56) | 0.33 (0.29) | 0.15 (0.13) |
|  | 250 | 5 | 0.65 (0.34) | 0.6 (0.78) | 0.25 (0.18) |
|  | 500 | 5 | 0.3 (0.11) | 0.4 (0.38) | 0.15 (0.14) |
|  | 1000 | 10 | 0.75 (0.26) | 0.95 (0.60) | 0.7 (0.48) |
|  | 2000 | 2 | 0.5 (0.71) | 0.75 (0.35) | 0 (0) |
| *E. dilatata* | MHW | 10 | 0.33 (0.12) | 0.43 (0.60) | 0.45 (0.23) |
|  | 120 | 5 | 0.35 (0.14) | 0.2 (0.21) | 0.2 (0.27) |
|  | 250 | 5 | 0.45 (0.11) | 0.65 (0.34) | 0.5 (0.30) |
|  | 500 | 5 | 0.25 (0.00) | 1.4 (0.55) | 0.15 (0.22) |
|  | 1000 | 5 | 0.55 (0.27) | 1.1 (0.55) | 1.5 (0.71) |
|  | 2000 | 4, 2, 1 | 0.38 (0.25) | 0.75 (0.35) | 0.25 (NA) |

**Table S5**. Average length (mm) and estimated age for mussels assigned to each chloride treatment (n = 5). Mussel age was estimated by counting external annuli.

| **Species** | **Treatment (mg Cl^-^/L)** | **Average Length** | **Standard Deviation** | **Average Age** | **Standard Deviation** |
| --- | --- | --- | --- | --- | --- |
| *Lasmigona costada* | 0 | 88.6 | 9.7 | 16.9 | 2.8 |
|  | 120 | 90.1 | 5.0 | 15.3 | 2.9 |
|  | 250 | 87.0 | 10.8 | 17.0 | 2.9 |
|  | 500 | 91.8 | 4.5 | 16.2 | 3.3 |
|  | 1000 | 89.3 | 4.8 | 14.6 | 3.0 |
|  | 2000 | 88.3 | 6.5 | 15.0 | 1.9 |
|  | 3750 | 90.9 | 5.0 | 14.2 | 2.4 |
| *Eurynia dilatata* | 0 | 64.8 | 3.8 | 12.1 | 1.9 |
|  | 120 | 64.3 | 4.0 | 10.2 | 1.3 |
|  | 250 | 62.7 | 3.5 | 13.0 | 1.9 |
|  | 500 | 61.9 | 4.2 | 11.6 | 2.7 |
|  | 1000 | 62.4 | 3.5 | 10.6 | 1.8 |
|  | 2000 | 61.3 | 4.4 | 10.4 | 1.1 |
|  | 3750 | 61.6 | 3.8 | 14.2 | 1.9 |

**Table S6.** Mean observed time filtering in adult mussels exposed to sodium chloride with standard deviation. Each treatment contained five mussels except the 0, 120, and 1000 mg Cl-/L treatments for *Lasmigona costada* where *n* = 10.

|  |  | **Average Filtration Time (%)** | | | |
| --- | --- | --- | --- | --- | --- |
| **Species** | **Treatment**  **(mg Cl^-^/L)** | **Days 1 to 3** | **Days 4 to 7** | **Days 8 to 14** | **Days 15 to 28** |
| *Lasmigona costada* | 0 | 98.3 (2.9) | 95.8 (3.8) | 89 (11.4) | 94 (8.2) |
|  | 120 | 100 (0) | 95 (6.3) | 92 (2.7) | 86 (8.2) |
|  | 250 | 100 (0) | 95 (8.3) | 90 (10) | 94 (8.9) |
|  | 500 | 80 (0) | 85 (12.2) | 90 (7.1) | 96 (5.5) |
|  | 1000 | 81.7 (20.2) | 88 (15.1) | 98 (2.7) | 100 (0) |
|  | 2000 | 50.8 (11.3) | 75.4 (27.9) | 95 (11.2) | 80 (44.7) |
|  | 3750 | 30 (26.5) |  |  |  |
| *Eurynia dilatata* | 0 | 93.3 (7.6) | 95.8 (5.9) | 81 (8.2) | 99 (2.2) |
|  | 120 | 96.7 (5.8) | 96.7 (5.1) | 96 (5.5) | 98 (4.5) |
|  | 250 | 93.3 (11.5) | 95 (8.4) | 96 (5.5) | 96 (8.9) |
|  | 500 | 93.3 (11.5) | 91.7 (9.8) | 86 (11.4) | 98 (4.5) |
|  | 1000 | 100 (0) | 96.7 (5.2) | 92 (13) | 96 (5.5) |
|  | 2000 | 60.8 (40) | 80.4 (33.2) | 83.3 (23.6) | 80 (27.4) |
|  | 3750 | 53.3 (45.1) |  |  |  |

**Table S7**. Chi-square results comparing filtration observations between control and sodium chloride exposed mussels. All tests were completed with one degree of freedom. Each treatment contained five mussels, except for *Lasmigona costata* in 0, 120, and 1000 mg Cl^-^/L treatment, which contained 10 mussels. Numbers in bold are p-values that are <0.05. *Where chi-square tests could not be completed a Fisher Exact test was used.

| **Species** | **Treatment (mg Cl^-^/L)** | **Days 1 to 3** | | | **Days 1 to 7** | | | **Days 8 to 28** | | |
| --- | --- | --- | --- | --- | --- | --- | --- | --- | --- | --- |
|  |  | **p** | **X^2^** | **Power** | **p** | **X^2^** | **Power** | **p** | **X^2^** | **Power** |
| *Lasmigona costada* | 120 | 0.612 | 0.257 | 0.08 | 1 | 0 |  | 0.461 | 0.544 | 0.114 |
|  | 250 | 0.534 | 0.384 | 0.095 | 0.898 | 0.0164 | 0.052 | 0.848 | 0.0367 | 0.054 |
|  | 500 | 0.153 | 2.047 | 0.298 | 0.052 | 3.768 | 0.492 | 0.431 | 0.62 | 0.123 |
|  | 1000 | 0.325 | 0.969 | 0.166 | 0.285 | 1.145 | 0.188 | 0.371 | 0.799 | 0.145 |
|  | 2000 | **<0.001*** |  |  | <**0.001** | 14.648 | 0.969 | **0.007** | 7.166 | 0.763 |
|  | 3750 | **<0.001*** |  |  |  |  |  |  |  |  |
| *Eurynia dilatata* | 120 | 0.871 | 0.0262 | 0.053 | 0.91 | 0.0127 | 0.051 | 0.063 | 3.455 | 0.459 |
|  | 250 | 0.656 | 0.199 | 0.073 | 0.848 | 0.037 | 0.054 | 0.065 | 3.398 | 0.453 |
|  | 500 | 0.656 | 0.199 | 0.073 | 0.437 | 0.604 | 0.121 | 0.867 | 0.028 | 0.053 |
|  | 1000 | 0.973 | 0.00119 | 0.05 | 0.833 | 0.0445 | 0.055 | 0.37 | 0.804 | 0.146 |
|  | 2000 | **0.002*** |  |  | **0.004** | 8.197 | 0.816 | **0.008** | 6.944 | 0.749 |
|  | 3750 | **<0.001*** |  |  |  |  |  |  |  |  |

**Table S8**. Statistically significantly metabolites extracted from hemolymph of *Lasmigona costata* exposed to chloride, electrospray ionization (ESI) negative mode. Entities with no data base matches numbered as Unknown. FC represents fold change.

| **Compound** | **p** | **FC 120 mg/L vs Blank** | **FC 1000 mg/L vs Blank** | **Mass** | **Database Score** | **Retention Time (min)** | **Frequency Found** |
| --- | --- | --- | --- | --- | --- | --- | --- |
| (-)-trans-C75 | 2.04E-04 | -1.31 | -2.35 | 254.1519 | 85.78 | 8.40 | 29 |
| (±)12-HETE | 8.60E-03 | 1.56 | 3.43 | 320.2349 | 83.13 | 12.84 | 29 |
| (±)16-HETE | 1.72E-05 | 1.48 | 3.85 | 320.2358 | 84.9 | 12.38 | 29 |
| 10,11-Epoxy-3,7,11-trimethyl-2E,6E-tridecadienoic acid | 4.52E-05 | -4.62 | -4.72 | 312.1938 | 85.04 | 9.50 | 29 |
| 12,15-epoxy-13,14-dimethyleicosa-12,14-dienoic acid | 2.35E-03 | 14.61 | 42.34 | 350.2819 | 82.27 | 13.99 | 27 |
| 13(S)-HOTrE | 2.95E-04 | 5.08 | 5.91 | 294.2195 | 83.72 | 11.43 | 29 |
| 18-Oxooleate | 3.70E-05 | 4.10 | 4.95 | 296.2354 | 85.29 | 12.19 | 29 |
| 2,4-dimethyl-2E-tetradecenoic acid | 9.62E-06 | 1.93 | 6.73 | 254.2246 | 84.6 | 15.20 | 29 |
| 3'-Prenyl-2',6',beta-trihydroxy-4'-methoxychalcone | 1.12E-02 | 11.61 | 152.98 | 354.1496 | 61.49 | 15.20 | 22 |
| 3β-Hydroxy-23,24-bisnorchol-5-enic Acid | 7.59E-03 | 1.87 | 2.39 | 346.2509 | 84.36 | 12.87 | 29 |
| 5(S)-HETE lactone | 7.15E-03 | -1.07 | 2.70 | 302.2249 | 99.8 | 14.48 | 29 |
| 5-Pentadecylresorcinol | 1.16E-03 | 1.63 | 3.33 | 366.2768 | 82.33 | 13.55 | 29 |
| 5Z-octadecenoic acid | 7.60E-06 | 1.53 | 4.06 | 282.256 | 85.17 | 16.46 | 29 |
| 7Z,10Z-octadecadienoic acid | 1.99E-02 | 1.28 | 3.26 | 280.2405 | 86.09 | 15.52 | 29 |
| 9(S)-HEPE | 6.18E-03 | 1.43 | 4.00 | 318.2197 | 84.29 | 11.53 | 29 |
| 9(S)-HEPE Esi-11.9800005 | 1.36E-02 | 1.40 | 2.98 | 318.2202 | 85.23 | 11.98 | 29 |
| Acutilol A | 1.49E-03 | -1.26 | 2.68 | 304.2404 | 99.76 | 15.29 | 29 |
| all-trans-retinyl oleate | 1.23E-03 | 2.96 | 712.25 | 610.4969 | 69.6 | 15.50 | 18 |
| Asn Asp Tyr | 1.12E-02 | 30.23 | 48.25 | 410.1478 | 63.78 | 6.96 | 26 |
| Bicyclo Prostaglandin E1 | 3.46E-05 | 112.06 | 3812.45 | 336.2304 | 80.29 | 10.56 | 18 |
| Dihydroxyneurosporene/ OH-Chloroxanthin | 3.47E-04 | -2.57 | 508.50 | 634.4955 | 68.6 | 15.44 | 16 |
| Eicosapentaenoic Acid ethyl ester | 1.09E-02 | -1.26 | 2.56 | 330.2563 | 99.77 | 15.45 | 29 |
| Eicosapentaenoic Acid ethyl ester Esi-15.782004 | 1.76E-02 | 1.45 | 3.95 | 330.2565 | 84.59 | 15.78 | 29 |
| His Lys Gln | 3.96E-06 | 1.47 | 2.67 | 457.2261 | 75.37 | 9.60 | 29 |
| Janthitrem E | 2.00E-02 | 6.33 | 125.19 | 663.3722 | 46.36 | 14.16 | 23 |
| Lauryl hydrogen sulfate | 5.89E-03 | 1.14 | -3.40 | 266.1554 | 98.51 | 12.28 | 29 |
| LysoPE(0:0/16:0) | 5.17E-04 | 308.82 | 222.46 | 453.2854 | 82.79 | 11.76 | 22 |
| Malyngamide L | 1.31E-03 | 41.80 | 271.13 | 513.289 | 43.73 | 11.79 | 23 |
| Met-Asn-OH | 2.27E-24 | -1.00 | 6054.97 | 417.0811 | 50.14 | 16.35 | 9 |
| Momordicasaponin II | 1.66E-10 | -8.88 | 3920.21 | 1760.6888 | 60.86 | 3.97 | 12 |
| N,N-Diisopropyltryptamine | 5.55E-03 | -1.19 | -2.63 | 280.1714 | 70.17 | 13.86 | 29 |
| Nafoxidine | 3.96E-08 | 1.88 | 3.98 | 485.2573 | 68.9 | 10.70 | 29 |
| N'-Nitrosoanabasine | 1.06E-02 | 1.67 | -31.15 | 237.1128 | 67.09 | 9.78 | 25 |
| PA(P-18:0/14:1(9Z)) | 2.00E-03 | 1.04 | 508.58 | 630.4628 | 72.77 | 15.29 | 17 |
| PE(18:0/0:0) | 4.77E-03 | 2.12 | 3.36 | 481.3168 | 99.65 | 11.87 | 29 |
| PE(19:0/0:0) | 4.93E-03 | 2.22 | 3.26 | 541.3382 | 79.48 | 11.87 | 29 |
| PE(20:0/0:0) | 1.00E-02 | 2.16 | 4.05 | 509.3479 | 99.61 | 13.57 | 29 |
| PE(22:1(11Z)/0:0) | 1.46E-03 | 1.37 | 3.01 | 595.3849 | 99.52 | 14.15 | 29 |
| PE(22:1(11Z)/0:0) Esi-12.830996 | 1.35E-03 | -1.52 | -2.20 | 595.3834 | 76.24 | 12.83 | 29 |
| PE(22:1(11Z)/0:0) Esi-13.843996 | 4.01E-03 | 1.70 | 3.62 | 595.3846 | 76.31 | 13.84 | 29 |
| PE(O-16:0/0:0) | 1.59E-02 | 1.21 | 2.72 | 499.3266 | 80.63 | 10.74 | 29 |
| PE(O-18:0/0:0) | 9.53E-03 | 1.14 | 2.27 | 467.3376 | 99.36 | 12.22 | 29 |
| PE(O-18:0/0:0) Esi-12.217002 | 1.41E-02 | 1.20 | 2.22 | 527.359 | 99.22 | 12.22 | 29 |
| PE(O-18:1(9Z)/0:0) | 1.12E-02 | 7.06 | 14.15 | 465.3219 | 82.41 | 13.68 | 28 |
| PE(O-18:1(9Z)/0:0) Esi-13.911997 | 1.37E-02 | 6.73 | 13.48 | 465.322 | 82.1 | 13.91 | 28 |
| PE(O-20:0/0:0) | 8.28E-03 | 1.01 | 2.18 | 541.3743 | 99.39 | 12.88 | 29 |
| PE(O-20:0/0:0) Esi-12.876005 | 1.01E-02 | 1.02 | 2.22 | 541.3742 | 78.96 | 12.88 | 29 |
| PE(O-20:0/0:0) Esi-14.118002 | 3.18E-03 | 1.22 | 2.72 | 555.3898 | 99.61 | 14.12 | 29 |
| PE(P-16:0/0:0) | 8.52E-03 | 33.76 | 36.67 | 437.2906 | 83.53 | 12.19 | 26 |
| PE(P-18:0/0:0) | 1.98E-02 | 1.12 | 2.08 | 525.3427 | 99.3 | 11.10 | 29 |
| PE(P-20:0/0:0) | 1.66E-02 | 1.39 | 2.55 | 553.3741 | 78.39 | 12.54 | 29 |
| Pentachloroethane | 2.53E-05 | -2.63 | -13.90 | 235.8273 | 36.28 | 17.85 | 29 |
| PS(20:2(11Z,14Z)/0:0) | 2.05E-03 | 29.87 | 80.97 | 609.3246 | 64.72 | 11.87 | 26 |
| Rutamarin | 4.39E-06 | 1.05 | 3.40 | 356.1658 | 69.24 | 16.34 | 29 |
| Trichilin A | 1.19E-04 | 1.33 | 3.16 | 734.3126 | 61.04 | 16.35 | 29 |
| Unknown 1 | 2.86E-07 | -1.25 | -7.54 | 313.8436 |  | 17.82 | 29 |
| Unknown 2 | 3.39E-04 | -1.44 | -113.84 | 481.7564 |  | 17.81 | 26 |
| Unknown 3 | 1.13E-06 | -1.29 | -3.43 | 217.8615 |  | 18.02 | 29 |
| Unknown 4 | 6.60E-03 | -1.44 | -13.11 | 317.7862 |  | 18.03 | 28 |
| Unknown 5 | 2.06E-07 | -1.56 | -4.11 | 117.9357 |  | 18.65 | 29 |
| Unknown 6 | 1.50E-03 | 1.50 | 6.64 | 1008.7113 |  | 12.22 | 29 |
| Unknown 7 | 8.77E-05 | -1.98 | 1104.22 | 1036.7411 |  | 12.89 | 14 |
| Unknown 8 | 1.34E-04 | 5.27 | 2005.85 | 1513.9368 |  | 6.26 | 19 |
| Unknown 9 | 1.55E-04 | 2.16 | 1794.10 | 1514.2731 |  | 6.26 | 18 |
| Unknown 10 | 1.16E-04 | 4.59 | 1743.43 | 1513.6017 |  | 6.27 | 19 |
| Unknown 11 | 5.41E-06 | -4099.97 | -1796.26 | 549.3541 |  | 11.37 | 15 |
| Unknown 12 | 1.04E-02 | 1.26 | 2.21 | 517.3299 |  | 12.21 | 29 |
| Unknown 13 | 8.36E-03 | 1.34 | 2.18 | 519.3282 |  | 12.21 | 29 |
| Unknown 14 | 2.73E-05 | 2.16 | 4.30 | 451.3061 |  | 12.81 | 29 |
| Unknown 15 | 6.97E-04 | 2.54 | 4.78 | 451.3067 |  | 12.82 | 29 |
| Unknown 16 | 3.22E-05 | -1019.64 | -6.75 | 717.5054 |  | 14.72 | 23 |
| Unknown 17 | 1.35E-05 | -1385.35 | -4.79 | 717.5059 |  | 15.34 | 23 |
| Unknown 18 | 2.96E-03 | 1.32 | 3.47 | 595.3462 |  | 12.22 | 29 |
| Unknown 19 | 1.40E-04 | 1.18 | 3.45 | 595.3457 |  | 12.22 | 29 |
| Unknown 20 | 4.17E-03 | 1.50 | 4.48 | 481.353 |  | 13.10 | 29 |
| Unknown 21 | 2.76E-08 | 2.07 | 5471.90 | 759.3151 |  | 4.36 | 12 |
| Unknown 22 | 4.71E-09 | 8285.86 | 8823.01 | 763.5515 |  | 18.03 | 19 |
| Unknown 23 | 1.35E-02 | 1.47 | 3.06 | 621.4008 |  | 14.45 | 29 |
| Unknown 24 | 3.51E-06 | 2.09 | 4179.03 | 842.2394 |  | 5.75 | 14 |
| Unknown 25 | 2.75E-03 | 18.10 | 569.99 | 946.6735 |  | 12.84 | 22 |
| α-Linolenic Acid | 6.92E-04 | -1.10 | 4.27 | 278.2247 | 86.07 | 14.64 | 29 |

**Table S9**. Statistically significantly metabolites extracted from hemolymph of *Lasmigona costata* exposed to chloride, electrospray ionization (ESI) positive mode. Entities with no data base matches numbered as Unknown. FC represents fold change.

| **Compound** | **p** | **FC 120 mg/L vs Blank** | **FC 1000 mg/L vs Blank** | **Mass** | **Database Score** | **Retention Time (min)** | **Frequency Found** |
| --- | --- | --- | --- | --- | --- | --- | --- |
| 11Z-octadecen-9-ynoic acid | 1.24E-03 | 1.2 | 4.0 | 278.2249 | 98.92 | 14.52 | 30 |
| 13Z-Octadecenal | 7.81E-08 | -1.3 | -2.4 | 288.2413 | 76.42 | 8.03 | 30 |
| 3-(3'-Methylthio)propylmalic acid | 8.46E-04 | 40.0 | 94.8 | 222.056 | 47.62 | 16.61 | 26 |
| 3'-Deoxystreptomycin 6,3''-bis-phosphate | 2.37E-05 | 206.3 | 537.5 | 742.2284 | 56.6 | 17.49 | 24 |
| 5(S)-HETrE | 1.39E-03 | 3.0 | 7.0 | 322.2503 | 83.79 | 13.71 | 30 |
| 6-hydroxysphingosine | 7.43E-04 | -2.2 | -224.4 | 315.2773 | 85.42 | 10.62 | 26 |
| 9-Nonadecene | 6.29E-04 | 2.8 | -1.5 | 283.3231 | 85.63 | 11.82 | 30 |
| Alpha-D-Manp-(1 - 2)-a-D-Manp-(1 - 2)-a-D-Manp-(1 - 3)-b-D-Manp-(1 - 4)-D-GlcNAcp | 5.90E-05 | 2.6 | 3.8 | 729.2319 | 58.64 | 17.49 | 30 |
| Aquifoliunine EIV | 8.64E-06 | 456.7 | 652.0 | 890.2689 | 33.97 | 16.70 | 23 |
| Bis(5-hydroxynoracronycine) | 1.55E-04 | 69.9 | 369.9 | 668.2133 | 56.16 | 16.61 | 23 |
| Camptothecin | 2.43E-06 | 1.4 | 4.0 | 370.0945 | 60.88 | 15.65 | 30 |
| Camptothecin Esi+16.609993 | 9.45E-03 | 4.5 | 11.7 | 370.0925 | 59.91 | 16.61 | 29 |
| Citbismine D | 2.71E-05 | 135.6 | 1439.4 | 744.2289 | 62.81 | 17.49 | 20 |
| Epimedoside E | 8.99E-04 | 22.7 | 45.6 | 816.2524 | 57.72 | 16.56 | 28 |
| Gibberellin A100 | 6.62E-04 | 1.1 | 245.4 | 375.179 | 67.72 | 6.03 | 22 |
| Isorhamnetin 3-rhamnosyl-(1-2)-galactoside-7-glucoside | 2.49E-04 | 3.3 | 3.9 | 803.2549 | 38.91 | 16.56 | 30 |
| N-(2R-Hydroxypentacosanoyl)-2S-amino-1,3S,4R-octadecanetriol | 2.19E-04 | 10.8 | 27.4 | 697.6551 | 85.21 | 16.17 | 29 |
| N-Hexadecanoylpyrrolidine | 3.70E-03 | 1.3 | -50.7 | 326.3279 | 84.49 | 15.19 | 26 |
| PE(16:0/19:1(9Z)) | 9.36E-03 | 145.6 | 16.2 | 731.5468 | 68.55 | 18.06 | 25 |
| PE(22:4(7Z,10Z,13Z,16Z)/19:1(9Z)) | 8.94E-06 | 3322.8 | 649.8 | 807.5773 | 65.59 | 18.07 | 22 |
| PE(O-18:0/20:3(8Z,11Z,14Z)) | 4.62E-05 | 16.8 | -26.1 | 777.568 | 65.53 | 18.01 | 26 |
| PE(P-18:0/0:0) | 2.08E-03 | 3.2 | 9.0 | 465.3223 | 98.52 | 13.72 | 30 |
| PE(P-18:0/0:0) Esi+13.712004 | 5.59E-03 | 2.8 | 8.5 | 465.3218 | 82.1 | 13.71 | 30 |
| PE(P-18:1(9Z)/18:0) | 2.22E-05 | -332.5 | -4248.5 | 729.5675 | 66.64 | 18.04 | 19 |
| PE(P-20:0/17:0) | 1.34E-04 | 2.4 | -107.1 | 785.5922 | 95.27 | 18.05 | 27 |
| PE(P-20:0/19:1(9Z)) | 4.90E-08 | 10.5 | -496.6 | 793.5982 | 87.86 | 18.07 | 24 |
| Phalluside-1 | 7.96E-05 | 5.6 | 74.3 | 725.5432 | 68.28 | 16.01 | 29 |
| Polyporusterone G | 7.58E-04 | 1.3 | 2.0 | 477.3449 | 72.6 | 17.14 | 30 |
| Pyrophaeophorbide a | 6.00E-03 | 2.9 | 12.5 | 534.2613 | 98.94 | 16.44 | 30 |
| Pyropheophorbide a | 3.46E-03 | 3.7 | 17.6 | 534.2617 | 98.51 | 16.43 | 30 |
| Retinoic acid | 6.49E-03 | 1.5 | 5.1 | 300.2092 | 85.06 | 12.05 | 30 |
| TG(17:1(9Z)/20:5(5Z,8Z,11Z,14Z,17Z)/22:5(7Z,10Z,13Z,16Z,19Z))[iso6] | 3.03E-04 | -4.6 | 265.6 | 938.7313 | 0 | 7.30 | 23 |
| Tumonoic Acid A | 3.02E-05 | -1.2 | -2.2 | 339.241 | 82.07 | 10.58 | 30 |
| Unknown 26 | 2.46E-05 | 2.9 | 5.1 | 775.2234 |  | 16.56 | 30 |
| Unknown 27 | 6.61E-03 | 1.3 | 2.2 | 991.7298 |  | 16.26 | 30 |
| Unknown 28 | 3.56E-03 | 1.1 | 7.9 | 1138.414 |  | 5.97 | 30 |
| Unknown 29 | 1.07E-07 | 7.5 | 8304.9 | 1228.179 |  | 10.27 | 17 |
| Unknown 30 | 1.21E-09 | -11.0 | 2471.4 | 1228.324 |  | 10.27 | 16 |
| Unknown 31 | 2.59E-07 | 2.3 | 5110.4 | 2149.818 |  | 10.27 | 17 |
| Unknown 32 | 9.30E-08 | 1.9 | 6694.1 | 2149.566 |  | 10.27 | 17 |
| Unknown 33 | 5.64E-08 | 14.1 | 11993.6 | 1228.466 |  | 10.27 | 18 |
| Unknown 34 | 5.26E-07 | 1.6 | 3590.4 | 1228.036 |  | 10.28 | 17 |
| Unknown 35 | 1.75E-04 | 1.0 | 2.3 | 1010.66 |  | 17.34 | 30 |
| Unknown 36 | 7.99E-05 | 1.2 | 2.3 | 1005.707 |  | 17.35 | 30 |
| Unknown 37 | 7.17E-05 | 1.1 | 2.3 | 1010.662 |  | 17.39 | 30 |
| Unknown 38 | 5.19E-03 | 1.3 | 2.5 | 1010.659 |  | 17.47 | 30 |
| Unknown 39 | 3.16E-08 | 8.7 | 15936.0 | 1858.772 |  | 5.35 | 15 |
| Unknown 40 | 4.43E-07 | 8.5 | 6473.8 | 1858.774 |  | 5.36 | 17 |
| Unknown 41 | 4.59E-03 | 8.1 | 260.6 | 1352.304 |  | 5.85 | 22 |
| Unknown 42 | 7.94E-03 | 1.9 | 211.1 | 1352.526 |  | 5.85 | 20 |
| Unknown 43 | 3.58E-03 | 1.3 | 6.7 | 1142.012 |  | 5.86 | 30 |
| Unknown 44 | 5.70E-03 | 1.2 | 6.7 | 1142.013 |  | 5.86 | 30 |
| Unknown 45 | 3.75E-03 | 1.2 | 7.4 | 2853.526 |  | 5.86 | 30 |
| Unknown 46 | 3.51E-03 | 7.3 | 434.0 | 1352.415 |  | 5.86 | 20 |
| Unknown 47 | 2.77E-03 | 1.2 | 7.4 | 2853.525 |  | 5.86 | 30 |
| Unknown 48 | 2.67E-03 | 1.2 | 8.3 | 1138.214 |  | 5.96 | 30 |
| Unknown 49 | 2.04E-03 | 1.2 | 9.7 | 2845.021 |  | 5.97 | 30 |
| Unknown 50 | 9.37E-03 | 2.2 | 28.3 | 2844.534 |  | 5.97 | 29 |
| Unknown 51 | 2.34E-03 | 1.1 | 8.0 | 1138.814 |  | 5.97 | 30 |
| Unknown 52 | 1.69E-03 | 1.1 | 7.9 | 1139.014 |  | 5.97 | 30 |
| Unknown 53 | 1.29E-03 | -1.4 | 131.6 | 1514.276 |  | 6.27 | 25 |
| Unknown 54 | 1.53E-04 | 9.3 | 1717.9 | 1855.378 |  | 7.29 | 24 |
| Unknown 55 | 6.38E-04 | 1.2 | 393.1 | 1976.994 |  | 7.30 | 25 |
| Unknown 56 | 2.24E-06 | 69.1 | 23897.9 | 1745.21 |  | 7.30 | 20 |
| Unknown 57 | 5.05E-05 | -1.4 | 1848.6 | 1855.237 |  | 7.30 | 21 |
| Unknown 58 | 8.17E-04 | 1.2 | 125.1 | 2164.271 |  | 7.30 | 27 |
| Unknown 59 | 9.86E-05 | 1.5 | 1876.8 | 1913.408 |  | 7.31 | 22 |
| Unknown 60 | 1.29E-04 | 32.5 | 2502.6 | 2281.214 |  | 7.31 | 23 |
| Unknown 61 | 7.25E-04 | 5.6 | 263.8 | 1977.226 |  | 7.32 | 27 |
| Unknown 62 | 1.50E-04 | 13.0 | 2769.3 | 1773.841 |  | 7.32 | 23 |
| Unknown 63 | 1.70E-03 | -1.2 | 522.0 | 1743.34 |  | 7.32 | 23 |
| Unknown 64 | 3.26E-05 | 10.2 | 8010.7 | 1976.105 |  | 7.32 | 20 |
| Unknown 65 | 4.57E-04 | 3.7 | 575.1 | 1860.916 |  | 7.33 | 25 |
| Unknown 66 | 7.45E-06 | -5.2 | 1419.5 | 1771.067 |  | 7.33 | 20 |
| Unknown 67 | 1.87E-03 | 5.8 | 268.6 | 2374.689 |  | 7.90 | 24 |
| Unknown 68 | 2.10E-04 | 4.1 | 620.5 | 2523.783 |  | 7.92 | 23 |
| Unknown 69 | 2.01E-03 | 22.2 | 156.4 | 2788.116 |  | 7.93 | 25 |
| Unknown 70 | 8.22E-03 | -1.3 | 82.7 | 2787.854 |  | 7.94 | 23 |
| Unknown 71 | 5.05E-04 | 2402.7 | 1.3 | 2117.419 |  | 8.15 | 16 |
| Unknown 72 | 3.40E-03 | 2.1 | -70.1 | 2221.598 |  | 8.18 | 26 |
| Unknown 73 | 4.23E-05 | 1.8 | 5.5 | 2704.001 |  | 9.16 | 30 |
| Unknown 74 | 3.49E-04 | 1.0 | 3.5 | 2704.257 |  | 9.17 | 30 |
| Unknown 75 | 1.67E-07 | 1.1 | 2441.9 | 1186.018 |  | 9.57 | 16 |
| Unknown 76 | 4.98E-03 | 1.7 | 4.7 | 361.3549 |  | 16.29 | 30 |
| Unknown 77 | 1.89E-07 | -1.2 | 4.3 | 469.2897 |  | 7.24 | 30 |
| Unknown 78 | 5.03E-03 | 2.6 | 167.5 | 557.166 |  | 15.66 | 21 |
| Unknown 79 | 6.64E-05 | 1.5 | 51.3 | 930.8795 |  | 7.35 | 29 |
| Unknown 80 | 2.49E-04 | 1.4 | 378.4 | 811.8427 |  | 7.29 | 23 |
| Unknown 81 | 1.38E-07 | 2.0 | 5.7 | 629.1828 |  | 16.61 | 30 |
| Unknown 82 | 2.10E-04 | 1.1 | 2.6 | 462.1236 |  | 14.42 | 30 |
| Unknown 83 | 2.43E-04 | 1.0 | 2.5 | 462.1228 |  | 14.42 | 30 |
| Unknown 84 | 6.57E-03 | 1.3 | 2.1 | 513.3561 |  | 17.15 | 30 |
| Unknown 85 | 7.30E-04 | 87.5 | 252.2 | 782.1785 |  | 16.54 | 23 |
| Unknown 86 | 3.76E-04 | 3.4 | 3.9 | 805.254 |  | 16.55 | 30 |
| Unknown 87 | 6.64E-07 | 1.3 | 4.3 | 555.168 |  | 15.66 | 30 |
| Unknown 88 | 2.75E-06 | 2.3 | 5.3 | 703.2015 |  | 17.49 | 30 |
| Unknown 89 | 7.43E-04 | 1.4 | 2.5 | 583.199 |  | 15.66 | 30 |
| Unknown 90 | 8.32E-05 | 2.3 | 1149.5 | 939.1004 |  | 7.26 | 22 |
| Unknown 91 | 1.10E-04 | 2.5 | 3.6 | 731.233 |  | 17.48 | 30 |
| Unknown 92 | 3.36E-05 | 3.3 | 5.6 | 778.2231 |  | 16.55 | 30 |
| Unknown 93 | 2.43E-05 | 2.2 | 3.7 | 657.2182 |  | 16.61 | 30 |
| Unknown 94 | 1.50E-04 | 2.4 | 5.4 | 706.1576 |  | 17.49 | 30 |
| Unknown 95 | 1.19E-06 | 1.2 | 4.2 | 536.1426 |  | 15.66 | 30 |
| Unknown 96 | 2.79E-06 | 2.3 | 5.3 | 701.2028 |  | 17.49 | 30 |
| Unknown 97 | 6.83E-03 | 17.3 | 34.4 | 634.1413 |  | 16.61 | 27 |
| Unknown 98 | 7.16E-03 | 4.2 | 26.5 | 630.5106 |  | 18.07 | 29 |
| Unknown 99 | 6.10E-04 | 1.5 | 2.5 | 581.2 |  | 15.66 | 30 |
| Unknown 100 | 2.86E-04 | 681.7 | 185.5 | 892.2667 |  | 16.71 | 20 |
| Unknown 101 | 1.13E-03 | 3.0 | 730.7 | 913.4281 |  | 7.39 | 23 |
| Unknown 102 | 2.43E-07 | 3538.4 | 12263.5 | 654.5101 |  | 18.06 | 20 |
| Unknown 103 | 4.98E-03 | 366.0 | 55.3 | 880.2717 |  | 16.69 | 22 |
| Unknown 104 | 8.62E-04 | -7.1 | 62.5 | 988.1816 |  | 7.35 | 25 |
| Unknown 105 | 1.93E-05 | 3.0 | 5.2 | 777.2224 |  | 16.56 | 30 |
| Unknown 106 | 2.24E-07 | 2.0 | 5.7 | 627.1841 |  | 16.61 | 30 |
| Unknown 107 | 6.66E-03 | 1.3 | 2.1 | 996.6835 |  | 17.31 | 30 |
| Unknown 108 | 7.99E-05 | 2.1 | 3.6 | 655.2179 |  | 16.61 | 30 |
| Unknown 109 | 4.74E-04 | -2.2 | 255.7 | 910.2009 |  | 7.29 | 24 |
| Unknown 110 | 6.81E-03 | 1.3 | 2.2 | 996.6832 |  | 17.32 | 30 |
| Unknown 111 | 1.82E-04 | 156.2 | 226.3 | 780.1802 |  | 16.56 | 25 |
| zymosterol intermediate 1c | 3.36E-03 | -1.3 | -77.4 | 443.3725 | 55.8 | 15.73 | 26 |
| α-12(13)-EpODE | 5.18E-03 | 1.6 | 5.4 | 294.2192 | 28.83 | 12.46 | 30 |

**Table S10**. Pathways identified in mass profiler professional (MPP) associated with entities found in electrospray ionization (ESI) negative mode analysis (Species – *Homo Sapiens*).

| **Pathway Name** | **WikiPathway ID** | **Entities matched to Pathway** |
| --- | --- | --- |
| Selenium Micronutrient Network | WP15_98241 | α-Linolenic Acid |
| BMAL1-CLOCK,NPAS2 activates circadian gene expression | WP3355_101335 | α-Linolenic Acid |
| Activation of gene expression by SREBF (SREBP) | WP2706_101249 | α-Linolenic Acid |
| Oxylipin metabolism | WP4005_102359 | α-Linolenic Acid, (±)12-HETE, 13(S)-HOTrE |
| Metabolism of alpha-linolenic acid | WP4586_106804 | α-Linolenic Acid |
| Arachidonic acid metabolism | WP2650_101354 | (±)12-HETE |
| Incretin synthesis, secretion, and inactivation | WP2728_101229 | α-Linolenic Acid |
| G alpha (q) signalling events | WP4424_101713 | α-Linolenic Acid |
| G alpha (i) signalling events | WP4423_101714 | (±)12-HETE |
| Regulation of lipid metabolism by Peroxisome proliferator activated receptor alpha (PPARalpha) | WP2797_101600 | α-Linolenic Acid |
| Mitochondrial biogenesis | WP3331_101311 | α-Linolenic Acid |
| Oxylipin metabolism | WP4005_97819 | α-Linolenic Acid, (±)12-HETE, 13(S)-HOTrE |
| Transcriptional regulation of white adipocyte differentiation | WP2751_101450 | α-Linolenic Acid, (±)12-HETE, 13(S)-HOTrE |
| Class A-1 (Rhodopsin-like receptors) | WP4419_101720 | α-Linolenic Acid, (±)12-HETE |
| Activation of gene expression by SREBF (SREBP) | WP2706_103006 | α-Linolenic Acid |
| alpha-linolenic (omega3) and linoleic (omega6) acid metabolism | WP2724_101350 | α-Linolenic Acid |
| Circadian Clock | WP1797_101648 | α-Linolenic Acid |

**Table S11**. Pathways identified in mass profiler professional (MPP) associated with entities found in electrospray ionization (ESI) positive mode analysis (Species – *Homo Sapiens*).

| **Pathway Name** | **WikiPathway ID** | **Entities matched to Pathway** |
| --- | --- | --- |
| Differentiation Pathway | WP2848_102217 | Retinoic Acid |
| Vitamins A and D – action mechanisms | WP4342_98582 | Retinoic Acid |
| Cardiac Progenitor Differentiation | WP2406_89157 | Retinoic Acid |
| PPAR signaling pathway | WP3942_94205 | Retinoic Acid |
| Vitamin A and Carotenoid Metabolism | WP716_97560 | Retinoic Acid |
| Non-small cell lung cancer | WP4255_105677 | Retinoic Acid |
| Dopaminergic Neurogenesis | WP2855_87239 | Retinoic Acid |
| Nuclear Receptors | WP170_106664 | Retinoic Acid |
| Non-small cell lung cancer | WP4255_96916 | Retinoic Acid |
| Nuclear Receptors | WP170_71083 | Retinoic Acid |
| 4-hydroxytamoxifen, Dexamethasone, and Retinoic Acids Regulation of p27 Expression | WP3879_97670 | Retinoic Acid |
| Nuclear Receptors in Lipid Metabolism and Toxicity | WP299_96301 | Retinoic Acid |
| The citric acid (TCA) cycle and respiratory electron transport | WP2766_101141 | Retinoic Acid |
| 22q11.2 Deletion Syndrome | WP4657_107179 | Retinoic Acid |
| Phase I – Functionalization of compounds | WP4085_101753 | Retinoic Acid |
| Activation of anterior HOX genes in hindbrain development during early embryogenesis | WP3813_101509 | Retinoic Acid |
| Phase I – Functionalization of compounds | WP4085_104895 | Retinoic Acid |

**Table S12**. Pathways manually searched in Kyoto Encyclopedia of Genes and Genomes (KEGG) online database associated with entities found in electrospray ionization (ESI) positive and negative mode analysis (Species – *Pomacea canaliculata*).

| **Pathway Name** | **KEGG ID** | **Entities matched to Pathway** |
| --- | --- | --- |
| Alpha-Linolenic acid metabolism | pcan00592 | α-Linolenic Acid |
| Retinol mechanism | pcan00830 | Retinoic Acid |
| Fatty acid biosynthesis | pcan01040 | α-Linolenic Acid |
| Steroid biosynthesis | pcan00100 | Zymosterol 1c |


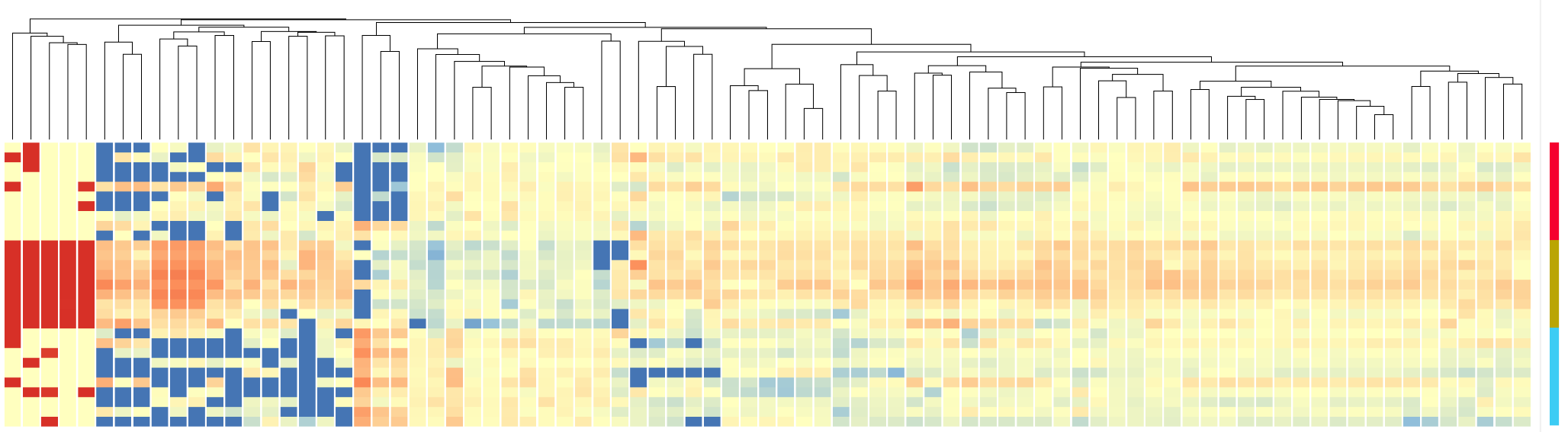

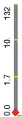

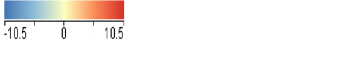


Treatment

Moderately Hard Water

120 mg/L Chloride

1000 mg/L Chloride

**Figure S1.** Heat map and hierarchical cluster analysis of negative polarity metabolites that were identified as significantly different between control mussels and those exposed to 120 and 1000 mg Cl^-^/L. Each row represents a replicate for each of the three treatments. Each column represents the abundance of a particular metabolite (red – high abundance, blue – low abundance).


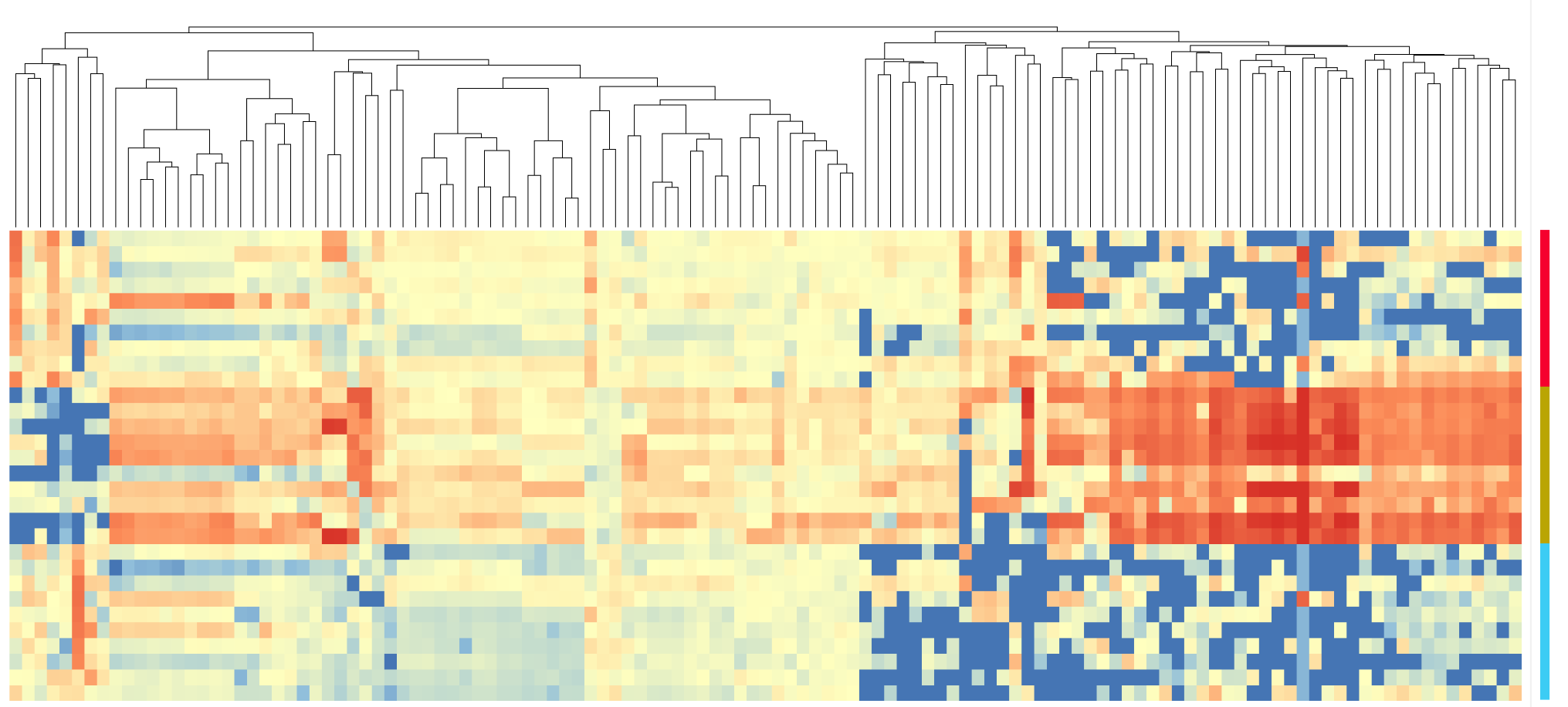


Moderately Hard Water

120 mg/L Chloride

1000 mg/L Chloride

Treatment


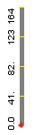

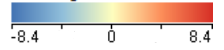


**Figure S2.** Heat map and hierarchical cluster analysis of positive polarity metabolites that were identified as significantly different between control mussels and those exposed to 120 and 1000 mg Cl^-^/L. Each row represents a replicate of the three treatments. Each column represents the abundance of a particular metabolite (red – high abundance, blue – low abundance).
